# Dysbiosis-Associated Enteric Glial Cell Immune-Activation and Redox Imbalance Modulate Tight Junction Protein Expression in Gulf War Illness Pathology

**DOI:** 10.3389/fphys.2019.01229

**Published:** 2019-10-14

**Authors:** Diana Kimono, Sutapa Sarkar, Muayad Albadrani, Ratanesh Seth, Dipro Bose, Ayan Mondal, Yuxi Li, Amar N. Kar, Mitzi Nagarkatti, Prakash Nagarkatti, Kimberly Sullivan, Patricia Janulewicz, Stephen Lasley, Ronnie Horner, Nancy Klimas, Saurabh Chatterjee

**Affiliations:** ^1^Environmental Health and Disease Laboratory, Department of Environmental Health Sciences, University of South Carolina, Columbia, SC, United States; ^2^Department of Basic Medical Sciences, Nanjing Medical University, Nanjing, China; ^3^Department of Biological Sciences, University of South Carolina, Columbia, SC, United States; ^4^Department of Pathology, Microbiology and Immunology, University of South Carolina School of Medicine, Columbia, SC, United States; ^5^Department of Environmental Health Sciences, Boston University School of Public Health, Boston, MA, United States; ^6^Department of Cancer Biology and Pharmacology, University of Illinois College of Medicine at Peoria, Peoria, IL, United States; ^7^Department of Health Services Policy and Management, University of South Carolina, Columbia, SC, United States; ^8^Department of Clinical Immunology, Nova Southeastern University, Fort Lauderdale, FL, United States

**Keywords:** dysbiosis, peroxynitrite, RAGE, S100B, nitric oxide, p47 phox, SsnB, sodium butyrate

## Abstract

About 14% of veterans who suffer from Gulf war illness (GWI) complain of some form of gastrointestinal disorder but with no significant markers of clinical pathology. Our previous studies have shown that exposure to GW chemicals resulted in altered microbiome which was associated with damage associated molecular pattern (DAMP) release followed by neuro and gastrointestinal inflammation with loss of gut barrier integrity. Enteric glial cells (EGC) are emerging as important regulators of the gastrointestinal tract and have been observed to change to a reactive phenotype in several functional gastrointestinal disorders such as IBS and IBD. This study is aimed at investigating the role of dysbiosis associated EGC immune-activation and redox instability in contributing to observed gastrointestinal barrier integrity loss in GWI via altered tight junction protein expression. Using a mouse model of GWI and *in vitro* studies with cultured EGC and use of antibiotics to ensure gut decontamination we show that exposure to GW chemicals caused dysbiosis associated change in EGCs. EGCs changed to a reactive phenotype characterized by activation of TLR4-S100β/RAGE-iNOS pathway causing release of nitric oxide and activation of NOX2 since gut sterility with antibiotics prevented this change. The resulting peroxynitrite generation led to increased oxidative stress that triggered inflammation as shown by increased NLRP-3 inflammasome activation and increased cell death. Activated EGCs *in vivo* and *in vitro* were associated with decrease in tight junction protein occludin and selective water channel aquaporin-3 with a concomitant increase in Claudin-2. The tight junction protein levels were restored following a parallel treatment of GWI mice with a TLR4 inhibitor SsnB and butyric acid that are known to decrease the immunoactivation of EGCs. Our study demonstrates that immune-redox mechanisms in EGC are important players in the pathology in GWI and may be possible therapeutic targets for improving outcomes in GWI symptom persistence.

## Introduction

Gastrointestinal disturbances are one of the most commonly reported chronic symptoms among veterans who returned from the Persian Gulf war of 1990–1991 (Murphy et al., 1999; Dunphy et al., 2003; Koch and Emory, 2005; White et al., 2016). About 14% of veterans who suffer from Gulf War illness (GWI) complain of some form of gastrointestinal (GI) problems such diarrhea, pain and gas etc. (Dunphy et al., 2003; Koch and Emory, 2005). According to Coker et al. (1999), the most commonly reported gastrointestinal issues reported among United States and British Gulf war (GW) veterans were diarrhea, vomiting, and stomach problems. A study by Dunphy et al. (2003) showed veterans of the Persian GW presented with diarrhea and had rectal hypersensitivity as did Zhou et al. (2018) who reported increased somatic hypersensitivity and pain among some GW veterans with GI issues.

Although the veterans report these symptoms, the prospective study by Koch and Emory (2005) did not find any significant clinical markers of disease pathology in blood or intestine tissue of deployed participants. Similarly, one of our own studies which reported metabolic reprogramming in liver as a result of leaky gut and endotoxemia did not find any biochemical markers of liver damage or altered metabolism in a mouse model of GWI. This was surprising because we had had previously shown that exposure to GW theater chemicals resulted in an alteration of gut microbiome and concomitant TLR4 mediated gastrointestinal and neuroinflammation with endotoxemia (Alhasson et al., 2017; Seth et al., 2018). This elusive nature of GWI is a strong reason for further studying underlying mechanisms of this condition in order to obtain effective therapies.

Of emerging interest in inflammatory gastroenterology are enteric glial cells (EGC) which reside in close proximity with the neurons of the enteric nervous system. These cells are similar in structure and physiology to astrocytes of the brain but are not excitable (Gershon and Rothman, 1991; Bassotti et al., 2007; Ochoa-Cortes et al., 2016). Initially, the principal function of EGC was thought to be providing mechanical support to enteric neurons. However, recent studies have shown that these cells play an important role in regulating the gastrointestinal microenvironment through several mechanisms, which have been extensively reviewed (Bassotti et al., 2007; Yu and Li, 2014; Sharkey, 2015). EGC were found to significantly modulate gut homeostasis through release of growth factors (von Boyen et al., 2011; Hansebout et al., 2012; Grubisic and Gulbransen, 2017) cytokines and prostaglandins (Hansebout et al., 2012; Steinkamp et al., 2012; Yu and Li, 2014) but may also play a pathogenic role by contributing to nitrosative stress and proinflammatory cytokines when exposed to stressful or toxic stimuli in the gut. Moreover, studies have found that EGC have the ability to “sense” a change in microbiome from probiotic to pathogenic, possibly through toll like receptors (TLRs). A study by Turco et al. (2014), found that adhesive *E. Coli* seem to activate a TLR-S100β/RAGE-iNOS signaling pathway in human EGC, while probiotic lactobacillus did not. Another study found that when EGC were treated with lipopolysacharrides (LPS), there was activation of TLRs with a release of S100B and nitric oxide (NO) (Cirillo et al., 2009; Rosenbaum et al., 2016). In this reactive state, EGC produce proinflammatory cytokines and chemokines e.g., (IL-1β, TNF-α, MCP-1) and release of inducible NO which may contribute to oxidative stress in the gut (von Boyen et al., 2011; Yu and Li, 2014; Ochoa-Cortes et al., 2016).

In irritable bowel syndrome (IBS) and inflammatory bowel disease (IBD), it is well known that an altered microbiome plays a significant role in the pathogenesis of the disease (Menees and Chey, 2018). In IBS for example, patients were found to have a decrease in abundance of Bifidobacteria and Lactobacillus but an increased prevalence of pathogenic species like *Escherichia* spp., Shigellas, and several Clostridia (Distrutti et al., 2016). Furthermore, it has been observed that metabolic diseases e.g., diabetes and obesity also present with increased ratio of Firmicutes to Bacteriodetes (Conlon and Bird, 2014; Johnson et al., 2017). Studies concerning the mechanisms of these gastrointestinal diseases have found that change of EGC phenotype from homeostatic to pathogenic is a characteristic of these diseases (Cabarrocas et al., 2003; Linan-Rico et al., 2016; Chen et al., 2018). A study by Wang et al. reported a significantly increased expression of glial fibrillary acidic protein (GFAP), Tyrosine receptor kinase B and Substance P in the colon of IBS patients with a correlated increase in intestinal inflammation (Wang et al., 2016). Other studies show that a loss in EGC resulted in poor gastrointestinal health characterized by loss of gut barrier integrity (Brown et al., 2016; Morales-Soto and Gulbransen, 2019).

Our previous research reported an altered microbiome in a murine model of GWI with increase in Firmicutes over Bacteriodetes and a decrease in several butyrogenic bacteria. This dysbiosis was accompanied by activation of TLR4, increased inflammation, a leaky gut, endotoxemia with release of damage associated molecular patterns (DAMPS) such as HMGB1 in gulf war chemical treated mice compared to controls (Alhasson et al., 2017; O’Callaghan et al., 2017; Seth et al., 2018). Interestingly, a recent study by Hernandez et al., showed that exposure to pyridostigmine bromide a known gulf war chemical exposure resulted in enteric neuronal and glial reactivity and inflammation (Hernandez et al., 2019).

This current study investigates the contribution of EGC in observed inflammatory phenotype which we and others have observed in GWI. We test the hypothesis that, the altered microbiome which results in increased pathogen associated molecular patterns (PAMPS) (e.g., LPS, flagellin and other immunostimulatory bacterial parts), leaky gut and increase in circulatory DAMPS (e.g., HMGB-1) in GW-chemical (Permethrin and pyridostigmine bromide) treated mice results in a reactive EGC phenotype compared to mice treated with vehicle control treated mice and mice co-exposed with GW chemicals and antibiotics. Through this reactive EGC phenotype intestinal cells such as enteric neurons and epithelial cells might be further affected leading to a vicious cycle of consistent proinflammatory state. This constant proinflammatory state of intestinal cells might answer the persistence of gastrointestinal, systemic and neuro inflammation in gulf war illness. The study uses a murine model of GWI and *in vitro* studies with EGCs and intestinal epithelial cells to elucidate possible mechanisms to explain this observed inflammation observed in GWI.

## Materials and Methods

Pyridostigmine bromide (PB), Permethrin (Per), Sodium Butyrate, Sparstolonin B (SsnB), Corticosterone, Neomycin trisulfate hydrate, Enrofloxacin, Apocynin (APO), Phenylboronic acid (FBA) were purchased from Sigma-Aldrich (St. Louis, MO, United States). Lipopolysaccharides (LPS), LPS-RS (TLR4 inhibibitor) were purchased from Cayman chemical company (Ann Arbor, MI, United States), Rat High mobility group box 1 protein (HMGB-1) Rat (rec) (His) was purchased from Chimirigen, Mediatech, Inc. (Manassas, VA, United States), Anti-TLR4, anti-flotillin-1, anti-S100B, anti-GFAP, anti-ASCII and anti-Caspase 1, anti-TLR5, anti-3NT, anti-GP91, anti-P47phox, anti-NOS 2, anti-HMGB-1 and anti-aquaporin-3 primary antibodies were purchased from Santa Cruz Biotechnology (Dallas, TX, United States), anti-claudin 2, anti-TLR2 and anti-occludin were purchased from Abclonal Technology (Woburn, MA, United States) was while anti-NLRP-3, anti-RAGE were purchased from Abcam (Cambridge, MA, United States). Fluorescence-conjugated (Alexa Fluor) secondary antibodies, ProLong Gold antifade mounting media with DAPI were purchased from Thermofisher Scientific (Grand Island, NY, United States), Apoptosis ApopTag^®^ Fluorecein *in situ* detection kit from Millipore (Billerica, MA, United States), The Pierce LAL Chromogenic Endotoxin Quantification Kit from Thermo Scientific (Waltham, MA, United States) and Griess reagent system from Promega corporation (Madison, WI). All other chemicals which were used in this study were purchased from Sigma unless otherwise specified. Paraffin-embedding of tissue sections on slides were done by AML laboratories (Baltimore, MD, United States).

### Animals

Adult wild-type male (C57BL/6J mice) were purchased from the Jackson Laboratories (Bar Harbor, ME, United States). Mice were implemented in accordance with NIH guideline for human care and use of laboratory animals and local IACUC standards. All procedures were approved by The University of South Carolina at Columbia, SC, United States. Mice were housed individually on 7090 Sani-Chip bedding from Teklad (Madison, WI, United States) and fed with 8904 irradiated chow diet from Teklad (Madison, WI, United States) at 22–24°C with a 12-h light/12-h dark cycle. All mice were sacrificed after animal experiments had been completed. Immediately after terminal anesthesia, mice’s small intestine was collected and dissected for further experiments, while fecal pellets were collected from the colon and immediately stored in sterile Eppendorf tubes for microbiome analysis. The tissues were fixed using 10% neutral buffered formalin. Distal segments of small intestines were used for the staining and visualizations.

### Rodent Model of Gulf War Illness (GWI)

Mice were exposed to Gulf War chemicals based on established rodent models of Gulf War Illness with some modifications (Zakirova et al., 2015; O’Callaghan et al., 2017). The treated wild-type mice group (GW) were dosed tri-weekly for 1 week with PB (2mg/Kg) and Permethrin (200 mg/kg) by oral gavage. After completion of PB and Permethrin dosages, mice were administered corticosterone intraperitoneally (i.p.) with a dose of 100μg/mice/day for 5 days of the week for 1 week to represent war stress. The dose of corticosterone was selected from the study which exposed mice to 200 mg/L of corticosterone through drinking water. The i.p. dose of corticosterone had similar immunosuppression as examined by low splenic T cell proliferation (data not shown). The vehicle control group (CONT) of mice received saline injections and vehicle for oral gavage (6% DMSO) in the same paradigm. Similarly, another group of mice (GW + AB) were exposed to PB/Permethrin and corticosterone as in above mentioned dosages along with antibiotics (Neomycin 45 mg/kg and Enrofloxacin 1mg/Kg) thrice per week for 2 weeks for intestinal decontamination and obtaining gut sterility, while another group (AB) were exposed to antibiotics (Neomycin, 45 mg/kg and Enrofloxacin 1 mg/Kg) for 2 weeks. A fifth group of mice was treated with PB/Permethrin and corticosterone, but with Sodium butyrate (10 mg/Kg) and Sparstolonin B (SsnB) 3 mg/Kg (GW + SsnB + BT). We have shown before that SSnB is a potent TLR4 antagonist and Butyrate decreases intestinal inflammation in GWI.

### Cell Culture

#### Enteric Glial Cell Culture

Immortalized rat EGC were obtained from ATCC^®^ (ATCCCRL-2690). Plated EGC were maintained in DMEM media supplemented with 10% FBS until treated. Cells were serum starved in DMEM supplemented with 1% FBS for 12 h and then exposed to vehicle control and chemicals. Cells were then treated with vehicle control-PBS (VEH), LPS (1 μg/mL), HMGB-1 (100 ng/mL), SsnB (10 μg/mL), Sodium butyrate (5 mM)and inhibitors FBA (100 μM) and Apocynin (100 μM) with either HMGB-1, LPS or antibiotics (neomycin and enrofloxacin cocktail) at different dilutions ranging from (1X to 1000X) (see Supplementary Figures S2–S7) for 24 h. After which the experiment was terminated and cells were harvested for mRNA extraction, gene expression analysis and protein expression studies. Nitric oxide production was estimated from culture fluids by measuring nitrite formation using the Griess assay.

#### Intestinal Epithelial Cell Culture

Immortalized rat intestinal epithelial cells (IEC-6) ATCC© CRL-1592, were obtained from ATCC. The cells were maintained in DMEM media supplemented with 10% FBS and 1x ITS until treated. Cells were serum starved in DMEM supplemented with 1% FBS for 12 h and then primed with LPS (100 ng/mL) for 12 h. Cells were then treated with culture fluids from EGC (above) for 24 h, then harvested for further analyses.

### Microbiome Analysis

Microbiome was analyzed from fecal pellets and luminal contents collected from animals after sacrifice and sent to Second Genome for 16S rRNA sequencing. Microbial analyses were performed from isolated nucleic acids using the MoBio PowerMag Microbiome kit (Carlsbad, CA, United States), according to manufacturer’s instructions. The microbiome data is in NCBI EBI under the accession number PRJEB19474.

### Laboratory Methods

#### Immunofluorescence Staining

Paraffin-embedded distal part of the small intestine sections were deparaffinized using a standard protocol. Epitope retrieval solution and steamer were used for epitope retrieval of sections. Primary antibodies such as anti- GFAP, anti-S100β, anti-NOS2, anti-NLRP-3, anti-ASCII, anti-GP91, anti-P47phox anti-TLR4, anti-Flotillin, anti-aquaporin3 were used at the recommended dilution (1:200). Species-specific secondary antibodies conjugated with Alexa Fluor (633-red and 488-green) were used at advised dilution (1:300). Finally, the stained sections were mounted using Prolong gold anti-fade reagent with DAPI. Sections were observed under–Olympus fluorescence microscope using 20X, 40X or 60X objective lenses, or under confocal microscopy using Leica SP-8 with LasX-10 software at magnification of 63X.

### Western Blot

Serum HMGB-1 levels were estimated from 35 μg of denatured mouse serum protein, while TLR2, 4 and 5 were estimated from 25 μg of denatured small intestine tissue by a Western Blot analysis following standard protocols. Briefly, serum was concentrated and then diluted 1:5. Tissue protein or serum protein was then separated on a Novex 4–12% bis-tris gradient gel and subjected to standard SDS-PAGE. The separated proteins were then transferred to a nitrocellulose membrane by a Trans-Blot Turbo transfer system. The membrane was then stained with Ponceau S, and then blocked with 5% BSA solution for 1 h, then incubated with primary antibody overnight at 4°C. Species-specific anti-IgG secondary antibody conjugated with HRP was used to tag primary antibody. ECL western blotting substrate was used to develop the blot The blot was then imaged using G:Box Chemi XX6 and subjected to densitometry analysis using Image J software.

#### Real-Time Quantitative PCR

Messenger RNA expression in small intestine and rat EGC was examined by quantitative real-time PCR analysis. Total RNA was isolated from each 15 mg small intestine tissue or 1 × 10^6^ EGC by homogenization in Trizol reagent (Invitrogen, Carlsbad, CA, United States) according to the manufacturer’s instructions and purified with the use of RNeasy mini kit columns (Qiagen, Valencia, CA, United States). cDNA was synthesized from purified RNA (1 μg) using iScript cDNA synthesis kit (Bio-Rad, Hercules, CA, United States) following the manufacturer’s standard protocol. Real-time qPCR (qRTPCR) was performed with the gene-specific primers using SsoAdvanced SYBR Green Supermix and CFX96 thermal cycler (Bio-Rad, Hercules, CA, United States). Threshold Cycle (Ct) values for the selected genes were normalized against respective samples internal control (18S). Each reaction was carried out in triplicates for each gene and for each sample. The relative fold change was calculated by the 2-ΔΔCt method. The sequences for the primers used for Real-time PCR are provided below in Table 1.

**TABLE 1:** Rat primer sequence.

Rat_IL-1β	Sense: CCTCGGCCAAGACAGGTCGC
	Antisense: TGCCCATCAGAGGCAAGGAGGA
Rat_NLRP-3	Sense: TGCATGCCGTATCTGGTTGT
	Antisense: ATGTCCTGAGCCATGGAAGC
Rat_TNF-α	Sense: CAACGCCCTCCTGGCCAACG
	Antisense: TCGGGGCAGCCTTGTCCCTT
Rat_ASCII	Sense: GGACAGTACCAGGCAGTTCG
	Antisense: GTCACCAAGTAGGGCTGTGT
Rat_Caspase 1	Sense: GACAGGTCCTGAGGCCAAAG
	Antisense: AAAAGTTCATCCAGCAATCCATTT
Rat_MCP 1	Sense: TAGCATCCACGTGCTGTCTC
	Antisense: CAGCCGACTCATTGGGATC
Rat_18S	Sense: GGATCCATTGGAGGGCAAGT
	Antisense: ACGAGCTTTTTAACTGCAGCAA
Rat_NOS2	Sense: AGCAGAGTTGGTGCAGAAGC
	Antisense: GGGAATAGCACCTGGGTTTT
Rat_Claudin1	Sense: AGGTCTGGCGACATTAGTGG
	Antisense: CGTGGTGTTGGGTAAGAGGT
Rat-ZO-1	Sense: GGAAATGTGTAAATCACCTGGAAGA
	Antisense: CCAAAGAACAGAAGACCACCAAC
mm_18S	Sense: TTCGAACGAACGTCTGCCCTATCAA
	Antisense: ATGGTAGGCACGGCGATA
mm_Claudin1	Sense: TTTCGCAAA GCACCGGGCATACA
	Antisense: GCCACTAATGTCGCCAGACCTGAAA
mm_ZO-1	Sense: CCACCTCTGTCCAGCTCTTC
	Antisense: CACCGGAGTGATGGTTTTCT
mm_TLR2	Sense: ACCAAGATCCAGAAGAGCCA
	Antisense: CATCACCCGGTCAGAAAACAA
mm-TLR4	Sense: GGAGTGCCCGCTTTCACCTC
	Antisense: ACCTTCCGGCTCTTGTGGAAGC
mm-TLR5	Sense: TGTAAAGTACTGGTGCCCGTGTGT
	Antisense: ACTGCGCAAACATTCTGCTGGC
mm-NOS-2	Sense: CGCTGGCTACCAGATGCCCG
	Antisense: GCCATAGCGGGCTTCCAGC

*The primer sequences are given as 5′-3′ orientation; Sense: Forward primer. Antisense: Reverse primer.*

### Endotoxin Level Detection by Litmus Amebocyte Lysate Assay

Bacterial endotoxins (EU/mL) were detecte din mouse stool samples for mice which were treated with vehicle control, gulf war chemicals, and mice co-exposed with gulf war chemicals and antibiotics using the Pierce LAL Chromogenic Endotoxin Quantification Kit according to the manufacturer’s instructions. Briefly, stool samples were obtained from mice and equal weights were homogenized in endotoxin free water. The supernatant was then collected, and heat inactivated at 70°C. This was then diluted 1:300 and endotoxins quantified.

### Nitrite Estimation by Griess Assay

Nitric oxide release was estimated from the cell culture fluids by measuring nitrite formation immediately after the experiment was terminated. Nitrite was measured using the Griess reagent system from Promega corporation (Madison, WI, United States) and experiments were performed according to manufacturer’s protocols.

### Tunel Assay

DNA fragmentation was detected using the ApopTag^®^ Fluorescein *in situ* detection kit from Millipore (Billerica, MA, United States) by following the manufacturer’s standard protocol.

### Statistical Analysis

All *in vivo* experiments were repeated three times (*N* = 3) with at least 3 mice per group (*n* = 9; data from each group of three mice were pooled). All *in vitro* and laboratory analysis experiments were repeated at least three or four times. The statistical analysis was carried out by analysis of variance (ANOVA) (see Supplementary Table S1 for F-statistics) and a Turkey’s HSD test to determine specific group differences. Further we performed an unpaired student *t*-test, using Graph pad prism software (GraphPad Software Inc., La Jolla, CA, United States). For all analyses ^∗^*P* < 0.05 was considered statistically significant and are marked as (^∗^).

## Results

### Altered Microbiome Is Associated With Increase in PAMPs and DAMPs in Gulf War Chemical Exposed Mice

**FIGURE 1:**
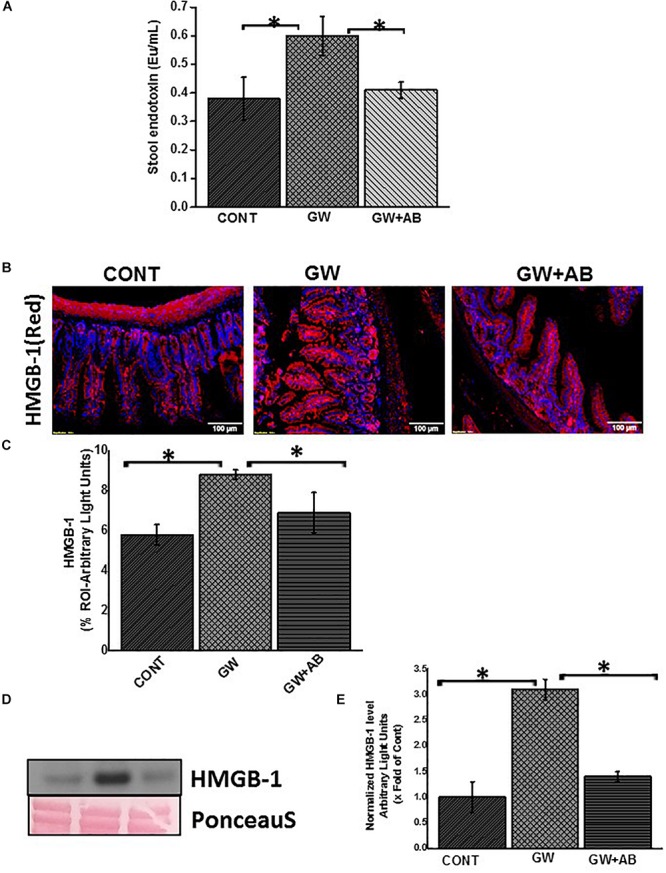
Altered microbiome associated increase PAMPS and DAMPS. **(A)** Stool endotoxin levels. Endotoxin levels in stool samples were determined by the LAL assay. Graph **(A)** show the levels of endotoxin in Endotoxin Units (Eu) in vehicle control (CONT, *n* = 9) treated mice, gulf war chemical exposed mice (GW, *n* = 9) and mice co-exposed with antibiotics (GW + AB, *n* = 9) (^∗^*P* < 0.05). **(B)** Expression of HMGB-1 in small intestine tissues. Expression of HMGB1 was assessed by immunofluorescence microscopy at (total Magnification 200X; scale bar 100 μm). Images show immunoreactivity in the distal part of the small intestine for vehicle control treated mice (CONT, *n* = 9), GW chemical treated mice (GW, *n* = 9) and mice co-exposed with gulf war chemicals and antibiotics (GW, *n* = 9). **(C)** Quantitative morphometric analysis of HMGB-1 immunoreactivity represented as arbitrary light units in the region of interest (% ROI) ^∗^*P* < 0.05. **(D)** Serum High mobility group box 1 (HMGB1) levels. Serum HMGB-1 levels were estimated by western blot analysis for mice treated with control (CONT, *n* = 3), Gulf war chemical exposed mice (GW, *n* = 5) and mice co-exposed to antibiotics and GW chemicals (GW + AB, *n* = 3). Ponceau red staining was used for normalization of protein. **(E)** Quantitative morphometric analysis of western blot bands normalized against total Ponceau. The *Y* axis shows HMGB-1/Ponceau S ratio (^∗^*P* < 0.05).

Studies have shown an association between altered microbiome and increase in endotoxin levels in serum or feces (Palone et al., 2016; Palone et al., 2018; Seth et al., 2018). In this study, using the LAL assay, we estimated the endotoxin levels (PAMPS e.g., LPS) in the stools of mice which were treated with GW chemicals in comparison with the controls and found that there was a significant increase in endotoxin levels of mice treated with GW chemicals compared to the controls (Figure 1A; *P* < 0.05). We further assessed the amount of HMGB-1 which was released in the small intestine (Figures 1B,C) using immunofluorescence microscopy and in the blood circulation (Figures 1D,E) using a western blot analysis for serum HMGB-1 levels in the circulation. These high amounts of DAMPS and PAMPS in the body will reach the EGC and cause persistent glial reactivity.

### Altered Microbiome (Dysbiosis) Correlates With an Increased Expression of GFAP While Gut Decontamination With Antibiotics Decreases GFAP in Intestinal Enteric Glial Cells

**FIGURE 2:**
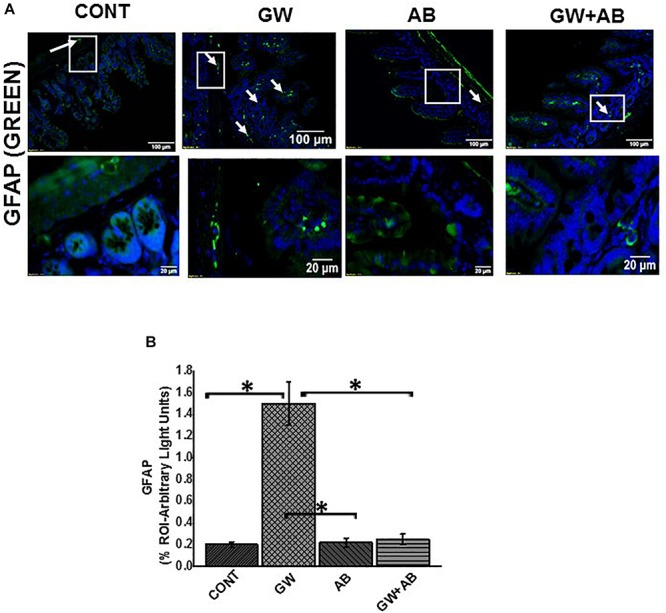
Altered microbiome induced change in EGC phenotype to a reactive phenotype. **(A)** Expression GFAP. Expression of GFAP was assessed by immunofluorescence microscopy at (top panel magnification 200X; scale 100 μm and bottom panel magnification 600X; scale 20 μm). Images show immunoreactivity of the distal part of the small intestine for vehicle control treated (CONT, *n* = 9), gulf war chemical treated mice (GW, *n* = 9), mice treated with only antibiotics (AB, *n* = 4) and mice co-exposed with gulf war chemicals and antibiotics (GW + AB, *n* = 9). **(B)** Quantitative morphometric analysis of GFAP immunoreactivity represented as arbitrary light units as observed in the region of interest (% ROI) (^∗^*P* < 0.05).

Enteric glial cells which are found in close proximity with enteric neurons are very abundant in the lamina propria, mucosa and sub mucosal regions of the small intestine (Bassotti et al., 2007). Using immunofluorescence microscopy, we found that there was a significant increase (*P* < 0.05) in GFAP expression in the small intestine of mice treated with GW chemicals (PB + BER) compared to the control group, and mice co-exposed to GW chemicals and antibiotics (Figures 2A,B). The increased expression of this protein has been associated with a reactive EGC phenotype in IBS and IBD (Akimoto, 2000; von Boyen et al., 2011).

### Altered Microbiome Correlates With a Reactive EGC Phenotype Through Activation of Toll-Like Receptors While Gut Decontamination via Antibiotic Usage Reversed Activation

**FIGURE 3:**
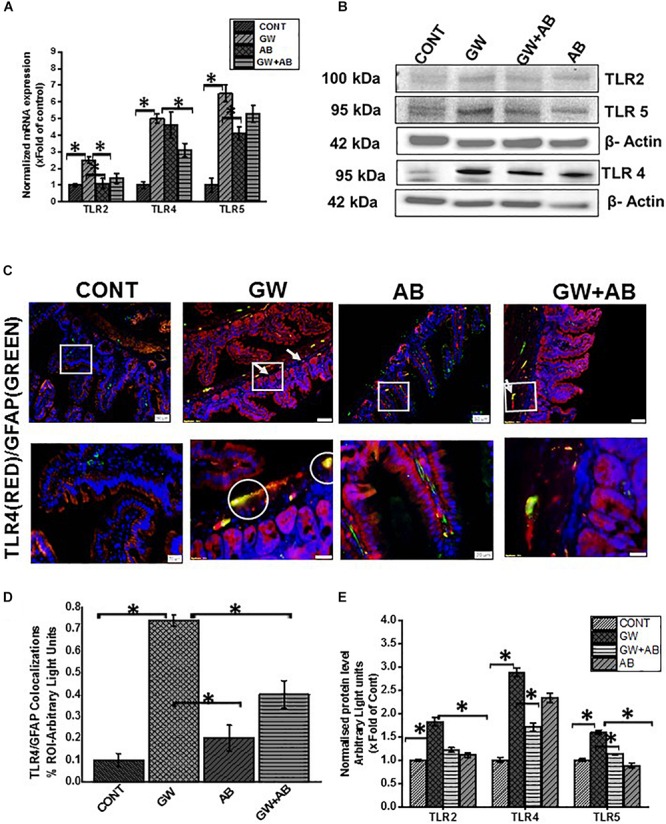
Expression of Toll-like receptors in small intestine and EGC. **(A,B)** General mRNA and protein expression levels of toll-like receptors TLR2, TLR4, and TLR5 in small intestine of mice treated with vehicle control (CONT, *n* = 9), gulf war chemical treated mice (GW, *n* = 9) and mice treated with antibiotics only (AB, *n* = 4) and mice co-exposed with GW chemicals and antibiotics (GW + AB, *n* = 9). mRNA expression was determined by RTqPCR, while protein expression was determined by western blot analysis. **(E)** Quantitative morphometric analysis of western blot bands normalized against β-actin The *Y* axis shows protein/β-actin ratio Results are expressed as mean ± SEM for *n* = 9 (^∗^*P* < 0.05). **(C)** Tissue level expression of TLR4 in EGC in small intestine. Expression of TLR4 in EGC was observed by in dual labeling of TLR4 and EGC cells marker GFAP via immunofluorescent microscopy visualized at (top panel magnification 200X; scale 100 μm and bottom panel magnification 600X; scale 20 μm) in small intestine tissues obtained from mice treated with vehicle control (CONT, *n* = 9); mice treated with GW chemicals (GW, *n* = 9) mice, mice treated with antibiotics only (*n* = 4) and co-exposed with GW chemicals and antibiotics (GW + AB, *n* = 3). **(D)** Quantitative morphometric analysis of immunoreactivity of GFAP/TLR4 (yellow) is represented as colocalizations events per field from randomly chosen microscopic fields (^∗^*P* < 0.05).

Our previous studies showed that the altered microbiome was associated with an activation of Toll like receptors such as TLR4 and TLR5 in GW chemical treated mice (Dyer and Walker, 1993; Seth et al., 2018). In this study we show that there was a significant increase mRNA (Figure 3A) and protein expression (Figures 3B,E) levels of TLR 2, 4, and 5 in mice which were exposed to gulf war chemicals (Permethrin and pyridostigmine bromide) compared to mice treated with only vehicle control and mice co exposed with GW chemicals and antibiotics (*P* < 0.05). We further detected a significant increased expression of TLR4 on EGC (TLR4/GFAP colocalizations) in GW chemical treated (GW) mice compared to Vehicle (CONT) and mice co-exposed with GW chemicals and antibiotics (GW + AB) (*P* < 0.05) (Figures 3C,D) by immunofluorescence microscopy.

### Altered Microbiome Associated Increased Expression of S100B in Reactive EGC Resulting in NOS-2 Expression While Antibiotic Usage for Depletion of Bacteria Reversed Such Activation

**FIGURE 4:**
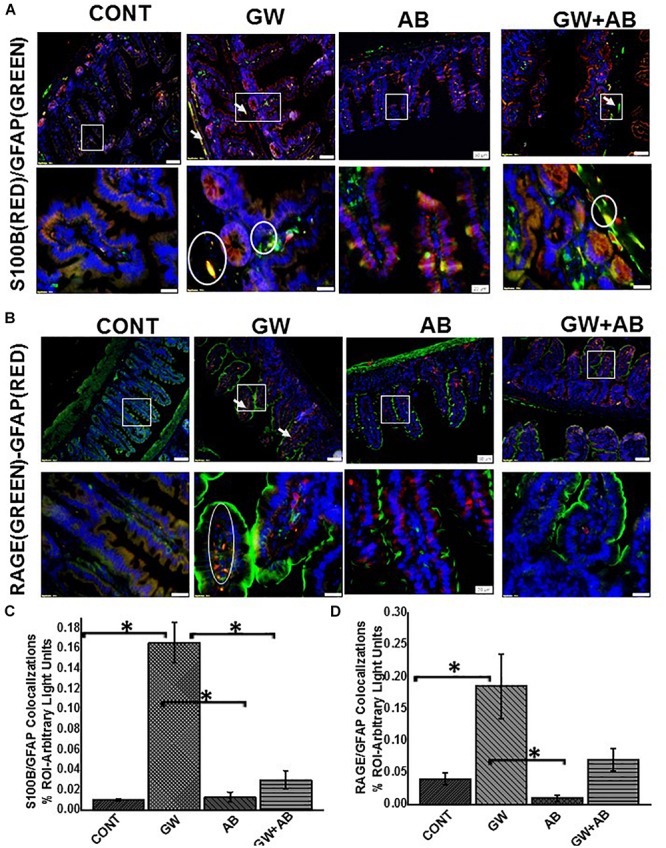
**(A)** Expression levels of S100B in EGC. Protein expression levels of S100B in EGC was determined by co-staining S100B with GFAP and assessed by immunofluorescence microscopy at (top panel magnification 200X; scale 100 μm and bottom panel magnification 600X; scale 20 μm). Images show immunoreactivity in distal part of the small intestine for vehicle control treated mice (CONT, *n* = 9), gulf war chemical treated mice (GW, *n* = 9) and gulf war chemical treated mice, mice treated with antibiotics only (AB, *n* = 4) and mice co-exposed with antibiotics (GW + AB, *n* = 9). **(C)** Quantitative morphometric analysis of immunoreactivity of GFAP/S100B (yellow) is represented as colocalizations events per field from randomly chosen microscopic fields (% ROI) (^∗^*P* < 0.05). **(B)** Expression of RAGE in EGC Protein expression levels of RAGE in EGC was determined by co-staining RAGE with GFAP and assessed by immunofluorescence microscopy at (top panel magnification 200X; scale 50 μm and bottom panel magnification 600X; scale 20 μm). Images show immunoreactivity in distal part of the small intestine for vehicle control treated mice (CONT, *n* = 9), gulf war chemical treated mice (GW, *n* = 9) mice treated with antibiotics only (AB, *n* = 4) and gulf war chemical treated mice co-exposed with antibiotics (GW + AB, *n* = 3). **(D)** Corresponding quantitative morphometric analysis of immunoreactivity of GFAP/RAGE (yellow) is represented as colocalizations events per field from randomly chosen microscopic fields (% ROI) (^∗^*P* < 0.05).

Using immunofluorescence microscopy, we found that there was a significant increase in the expression of S100B in GW chemical treated mice compared to mice treated with vehicle control and mice which were co exposed with GW chemicals and antibiotics (*P* < 0.05) (Figures 4A,C). We also found that there was a significant increase in RAGE expression in GW chemical treated mice in EGC by co-staining RAGE and GFAP (*P* < 0.05) compared to vehicle control treated mice. However, this increase was not significant for mice treated with both GW chemicals and antibiotics (Figures 4B,D).

**FIGURE 5:**
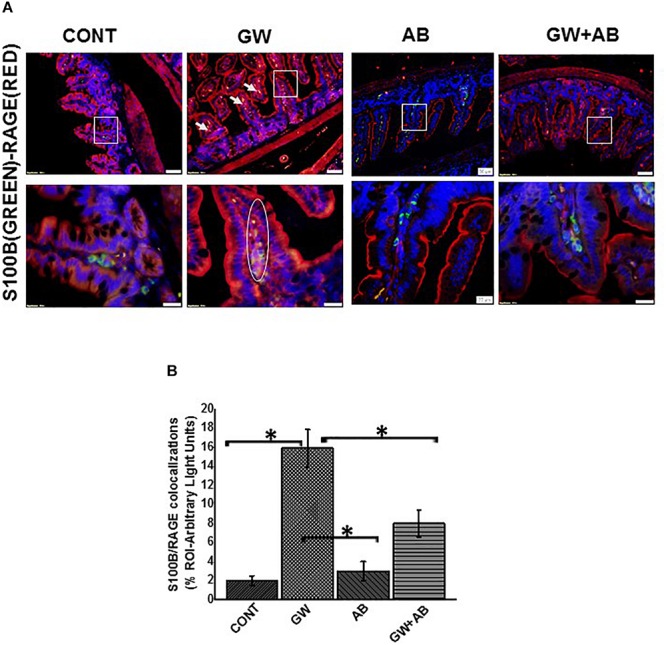
Formation of S100B/RAGE complex in small intestine. **(A)** S100B(Green)/RAGE(Red) complex formation expression in EGC in small intestine tissues. Protein expression levels were assessed by immunofluorescence microscopy of tissues at (top panel magnification 200X; scale 50 μm and bottom panel magnification 600X; scale 20 μm). Images show immunoreactivity the distal part of the small intestine for gulf war chemical treated mice (GW, *n* = 9), vehicle control (CONT, *n* = 9), mice treated with antibiotics only (*n* = 4) and mice co-exposed GW chemicals and antibiotics (GW + AB, *n* = 9). **(B)** Quantitative morphometric analysis of immunoreactivity for S100B/RAGE is represented as colocalization events per field for randomly chosen fields (% ROI) (^∗^*P* < 0.05).

**FIGURE 6:**
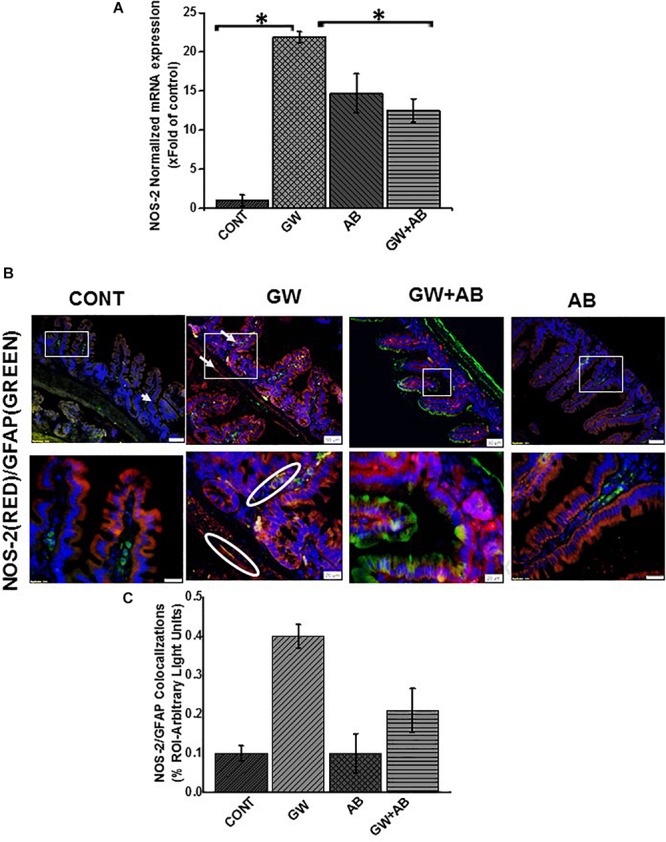
Activation of NOS-2 in small intestine. **(A)** NOS-2 mRNA expression in the small intestine of intestine of mice treated with vehicle control (CONT, *n* = 9), gulf war chemical treated mice (GW, *n* = 9), mice treated with antibiotics only (AB, *n* = 4) and mice co-exposed with GW chemicals and antibiotics (GW + AB, *n* = 9. ^∗^*P* < 0.05) was determined by RTqPCR. **(B)** Protein expression levels of NOS- 2 in enteric glial cells was determined by immunofluorescence microscopy of tissues and imaged at (top panel magnification 200X; scale 50 μm and bottom panel magnification 600X; scale 20 μm). **(C)** Quantitative morphometric analysis of immunoreactivity for GFAP/NOS-2 represented as colocalization events per field for randomly chosen fields (% ROI).

We then studied the interaction between RAGE and S100B using immunofluorescence microscopy assuming that a co-localization of these two proteins would suggest complex formation and aid interaction. We showed that there was significant increase (*P* < 0.05) in S100β/RAGE complex formation in GW chemical exposed mice (GW) and mice treated with vehicle control (CONT) or mice co-exposed with GW chemicals and antibiotics (GW + AB) (Figures 5A,B). In Figure 6A using RTqPCR we found that there was a significant increase in mRNA expression of inducible nitric oxide synthase in the small intestine of GW chemical treated mice and mice treated with vehicle control and mice co exposed with antibiotics and gulf war chemicals (*n* = 9, *p* < 0.05). Further, we showed that there was a marked increase in inducible nitric oxide synthase (NOS-2) expression in the intestine tissues of mice treated with GW chemicals (GW) compared to mice treated with vehicle control (CONT) and mice co-exposed with GW chemicals and antibiotics (GW + AB), although this increase was not significant (*P* = 0.075, and 0.11 respectively) (Figures 6B,C).

These results are evidence of activation of a TLR-S100β/RAGE-iNOS pathway in association to an altered microbiome *in vivo* as suggested by a decrease of activation following the use of antibiotics to ensure gut decontamination.

### Exposure to PAMPS (e.g., Lipopolysaccharides) and DAMPS (e.g., HMGB-1) Causes the Activation of TLR4-s100β/RAGE-NO Pathway in EGC

EGC can respond to an over balance in gut microorganisms by detecting PAMPS on/from the pathogen these bacteria such as cell wall, nucleic acid, flagella etc and mount an effective immune response through toll like receptors or NOD-like receptors (Turco et al., 2014).

**FIGURE 7:**
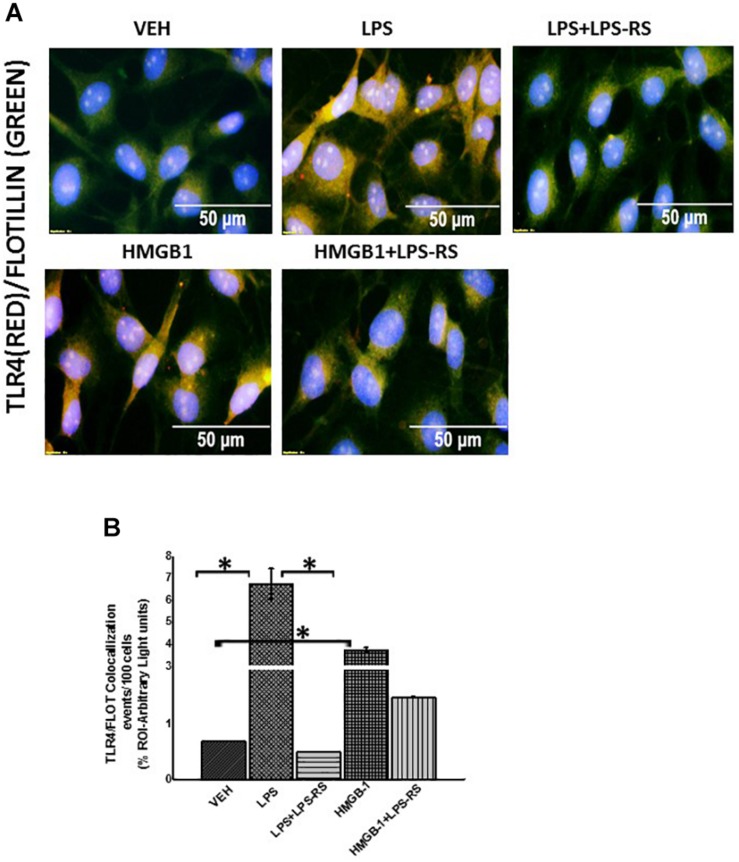
Exposure to DAMPS and PAMPS cause activation of TLR4 in EGC. **(A)** TLR4 activation in rat Enteric Glial Cells (EGC) (*n* = 6). Colocalization of TLR4 and Flotillin in EGC treated with either vehicle control (VEH) or LPS, LPS + LPS-RS, HMGB-1 or HMGB1 + LPS-RS determined by immunofluorescence microscopy at (magnification 400X; scale 50 μm). **(B)** Quantitative morphometric analysis of TLR4/Flotillin colocalizations between red and green immunoreactivity were determined for every 100 cells per field. Fields were chosen randomly, and colocalizations represented as immunoreactivity in the region of interest (% ROI) (^∗^*P* < 0.05).

**FIGURE 8:**
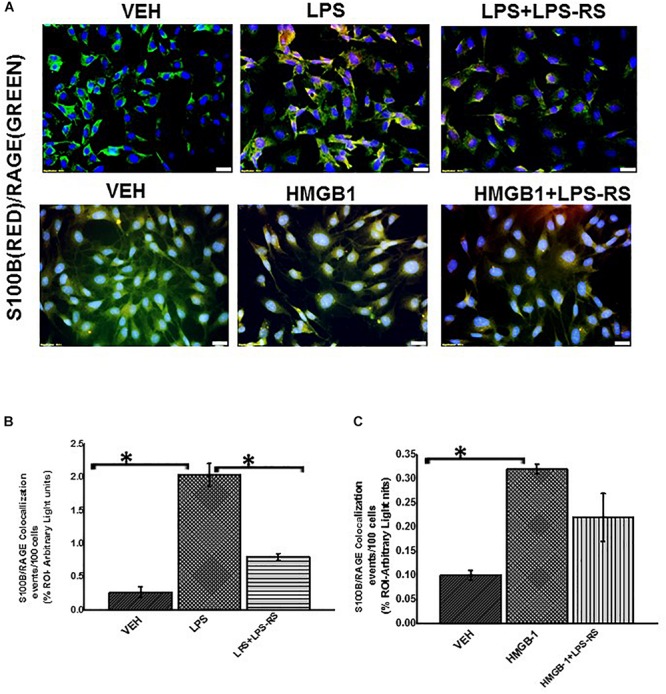
EGC exposed to LPS or HMGB-1 change to a reactive phenotype. **(A)** S100B/RAGE complex formation in rat Enteric Glial Cells (EGC) (*n* = 6). Colocalization of S100β and RAGE in EGC treated with either vehicle control (VEH) or LPS, LPS + LPS-RS, HMGB1 or HMGB-1 + HMGB-1 + LPS-RS was determined by immunofluorescence microscopy at (total magnification 400X and scale 20 μm). **(B,C)** Quantitative morphometric analysis of S100B/RAGE complex formation. Colocalizations between red and green immunoreactivity were determined for every 100 cells per field for randomly chosen fields and represented as immunoreactivity in the region of interest (% ROI) (^∗^*P* < 0.05).

Using immunofluorescence microscopy, we found that there was significant increase in TLR4 expression when we treated rat EGC with LPS or HMGB-1 (Figures 7A,B, *P* < 0.05). We also found an increase in S100β/RAGE complex formation in LPS and HMGB1 treated cells compared to cells treated with vehicle control (*P* < 0.05) (Figures 8A–C). However, the difference between expression of these receptors was not significantly different between the cells treated with HMGB1 alone compared to those treated with HMGB1 + LPS-RS to block the TLR4 receptor. This indicates that possibly, DAMPS like HMGB1 can trigger inflammatory pathways in EGCs via several other receptors apart from TLR4.

**FIGURE 9:**
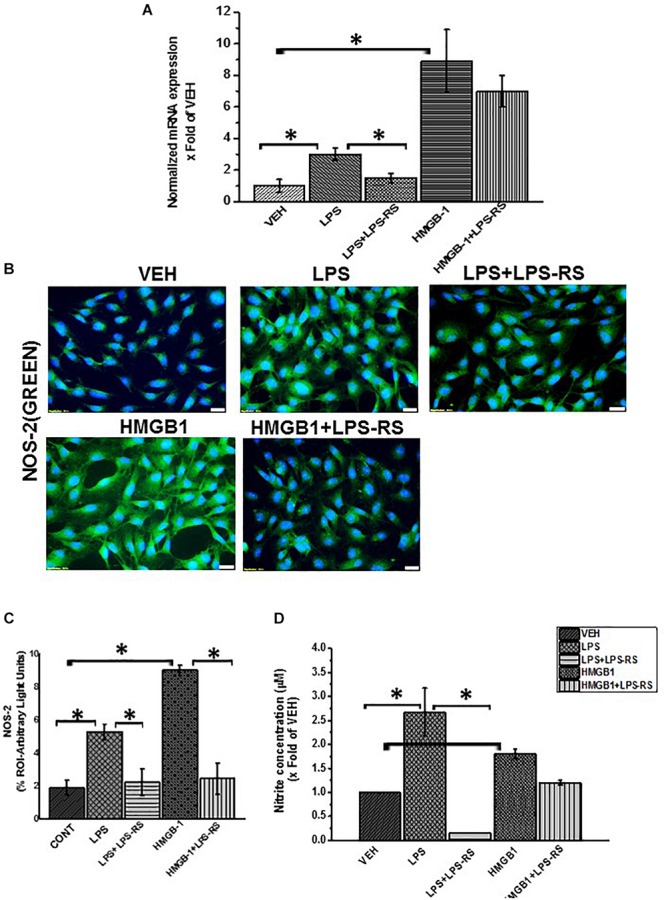
Activation of inducible nitric oxide synthase by LPS or HMGB-1. **(A)** mRNA expression of NOS-2 in EGC exposed to vehicle control (VEH), LPS, LPS + LPS-RS or HMGB-1 and HMGB-1 + LPS-RS (*n* = 6) expressed as x fold of the vehicle control. mRNA expression was determined by qRTPCR. **(B)** NOS-2 protein expression in the cells was detected by staining with green fluorescent antibody and counterstained with DAPI (blue) and viewed at (total magnification 400 μm and scale 20 μm). **(C)** Quantitative morphometric analysis of NOS-2 in rat EGC per 100 cells in different fields and represented as immunoreactivity in the region of interest (% ROI). **(D)** Nitrite concentration in EGC (*n* = 8). Nitric oxide production in EGC supernatants was estimated by Griess assay from freshly harvested supernatants. Nitrite concentration is reported as X fold increase over the vehicle control (VEH) (^∗^*P* < 0.05).

We further evaluated the activation of inducible nitric oxide synthase and release of nitric oxide in the rat EGC treated with LPS or HMGB1 (Figures 9A–D). We used RT q PCR to evaluate the expression of nitric oxide synthase in rat EGC (Figure 9A). Our results showed a significant increase in the expression of iNOS in cells treated with LPS or HMGB1 compared to vehicle control (*P* < 0.01). We also found that there was a significant increase in the protein expression of NOS-2 in LPS and HMGB-1 treated cells compared to cells treated with vehicle control only as evaluated by immunofluorescence microscopy (Figures 9B,C) (*n* = 3, *P* < 0.05). Finally, we investigated whether there was a release of nitric oxide by the cells (Figure 9D). We found that NO release was significantly increased LPS (2.6 fold) (^∗^*P* < 0.05), but only a marked increase in cells treated with HMGB1 (*P* = 0.07) compared to vehicle control treated cells. Together, these results indicate the activation of a TLR-S100B/RAGE pathway that subsequently led to the increased production of nitric oxide, especially in response to microbial PAMPS.

### Activation of NADPH Oxidase 2 (NOX-2) and Increased Peroxynitrite Formation in Small Intestine Following Dysbiosis and Its Reversal by Gut Sterility

**FIGURE 10:**
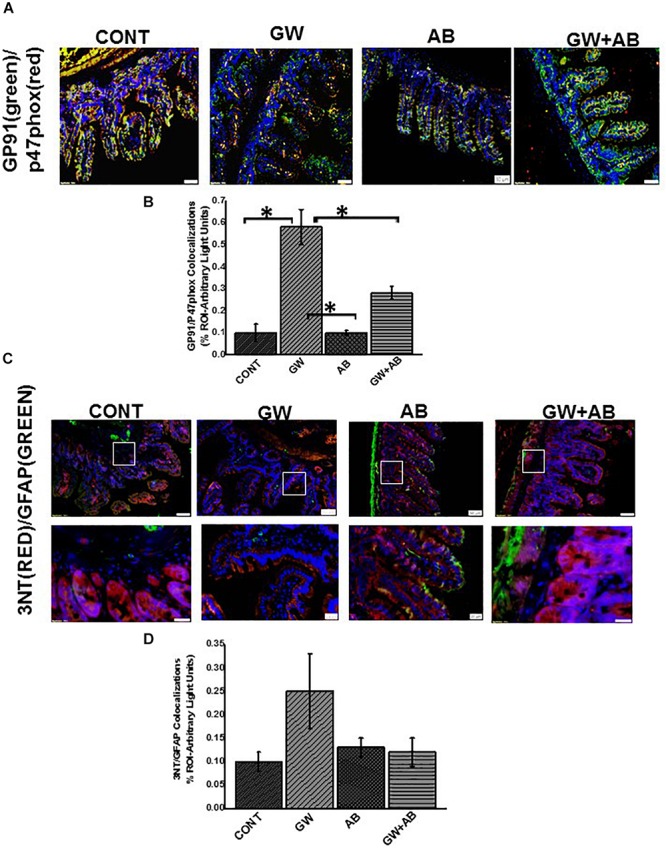
NADPH oxidase 2 and peroxynitrite mediated oxidative stress *in vivo*. **(A)** NOX-2 activation in small intestine assessed by immunofluorescence microscopy (*n* = 9) (at total magnification 200X and scale 50 μm). Activation of NOX 2 was studied through observing colocalizations between GP91phox (labeled with green fluorescent antibody) and P47 phox (labeled with red fluorescent antibody) subunits of the NADPH 2 oxidase complex resulting in a yellow region. Colocalizations were determined in small intestine tissues of CONT, GW, AB, and GW + AB chemical exposed mice. **(B)** Graphical representation of morphometric analysis of colocalization events of GP91phox and P47phox in the region of interest. **(C)** Immunoreactivity of 3-nitrotyrosine (3NT) in EGC assessed through observing colocalizations between GFAP (labeled with green fluorescent antibody) and 3NT phox (labeled with red fluorescent antibody) at top panel magnification 200X, scale 50 μm and bottom panel magnification 600X and scale 20 μm) Colocalizations were determined in small intestine tissues of CONT, GW, AB, and GW + AB chemical exposed mice. **(D)** Graphical representation of morphometric analysis of colocalization events of GFAP (green) and 3NT (red).

NADPH oxidase-2 with its subunits P47 phox, P67 phox, P22 align with GP91 in the membrane to form the NOX-2 membrane assembly. We detected the activation of NOX-2 by immunofluorescence dual labeling of GP91phox and P47phox subunits of the enzyme. We found a significant increase (*P* < 0.05) GP91phox-P47phox co-localization events in GW chemical treated mice (GW) compared to mice treated with vehicle (CONT, *n* = 9), mice exposed to antibiotics only (AB, *n* = 4) and mice co-exposed with GW chemicals and antibiotics (GW + AB, *n* = 9) (Figures 10A,B). Furthermore, we found a marked increase in peroxynitrite, an indicator of redox sensitive tyrosyl radicals in EGC of mice treated with GW chemicals compared to mice treated with either vehicle control or GW chemicals and antibiotics (Figures 10C,D) (^∗^*P* < 0.05).

### Activation of NOX-2 and Increased Peroxynitrite Formation in Rat EGC

**FIGURE 11:**
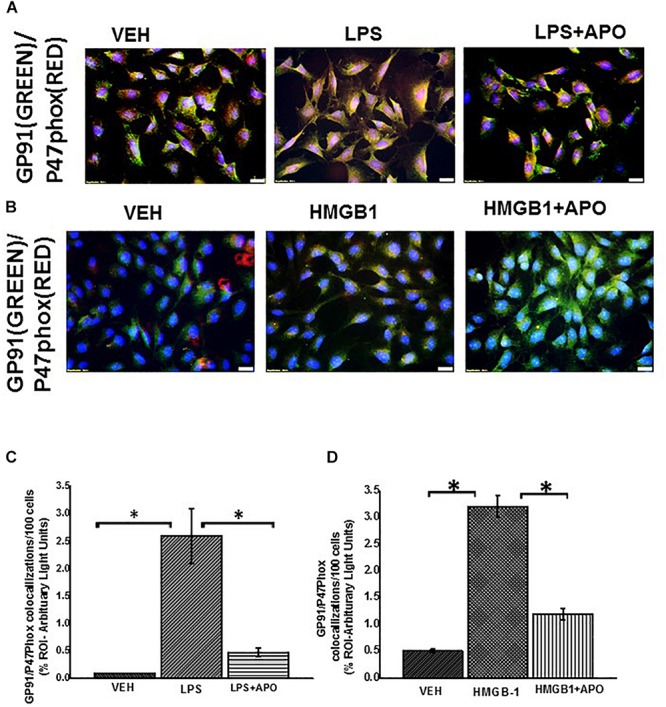
NADPH oxidase 2 activation in rat EGC. **(A,B)** Activation of NOX-2 in rat EGC (*n* = 6). Activation of NOX 2 was studied through observing colocalizations between GP91phox (labeled with green fluorescent antibody) and P47 phox (labeled with red fluorescent antibody) subunits of the NADPH 2 oxidase complex resulting in a yellow region. Colocalization (yellow) of GP91phox and P47phox was detected in vehicle VEH, LPS, LPS + apocynin (LPS + APO), HMGB-1, HMGB-1 + APO treated cells at total magnification 400X; scale 20 μm). **(C,D)** Morphometric analysis of GP91/p47phox colocalization events in rat EGC per 100 cells in different fields (^∗^*P* < 0.05).

Studies have showed that NADPH oxidases are activated in response to pathogenic stimuli in human EGC (Macchioni et al., 2017). We found that treatment of EGC with LPS or HMGB1 significantly increased their expression of NOX-2 (Figures 11A–D) (*P* < 0.05). This activation was observed by immunofluorescence microscopy by detecting co-localization events (per 100 cells) between two key subunits of the NOX-2 enzyme complex. One in the lipid membrane GP91phox (labeled with green secondary antibody) and P47phox (labeled with red antibody). We found a significant increase in these co-localizations in LPS or HMGB1 treated cells compared to vehicle control treated cells (VEH) and cells treated with LPS/HMGB1 and Apocynin (LPS + APO) a NADPH inhibitor (Apocynin blocks the transport of p47 phox to the membrane) (^∗^*P* < 0.05).

**FIGURE 12:**
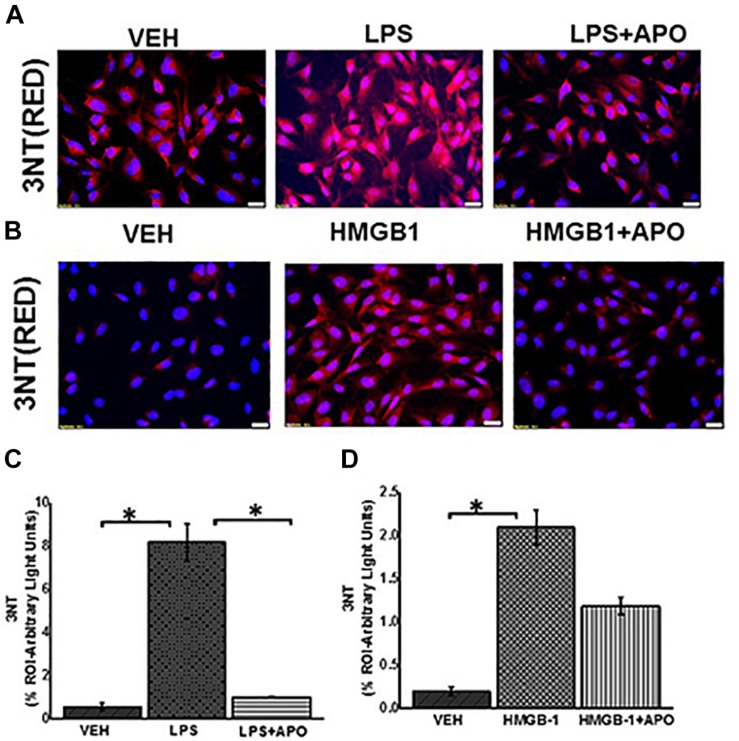
Peroxynitrite formation in EGC. **(A,B)** Expression of 3NT in rat EGC (*n* = 6). Immunoreactivity of 3NT was detected in vehicle (VEH), LPS, LPS + APO, HMGB-1 and HMGB-1 + APO treated cells by staining with red fluorescent antibody and counterstained with DAPI (blue) viewed at total magnification 400X; scale 20 μm). **(C,D)** Morphometric analysis of 3NT in rat EGC per 100 cells in different fields (^∗^*P* < 0.05).

NOX-2 induced superoxide and nitric oxide react rapidly to form peroxynitrite, an indicator of redox related formation of tyrosyl radical and subsequent formation of tyrosine nitration We also observed that there was a significant increase in formation of peroxynitrite (shown by increased 3- nitrotyrosine formation) in LPS or HMGB1 treated EGC compared to Vehicle control (VEH) treated and LPS or HMGB1 and apocynin (LPS + APO) or (HMGB1 + APO) (Figures 12A–D) treated EGC (*P* < 0.05, *n* = 6).

### Oxidative Stress Triggers Activation of NLRP-3 Inflammasome Which Results in Increased Inflammation

**FIGURE 13:**
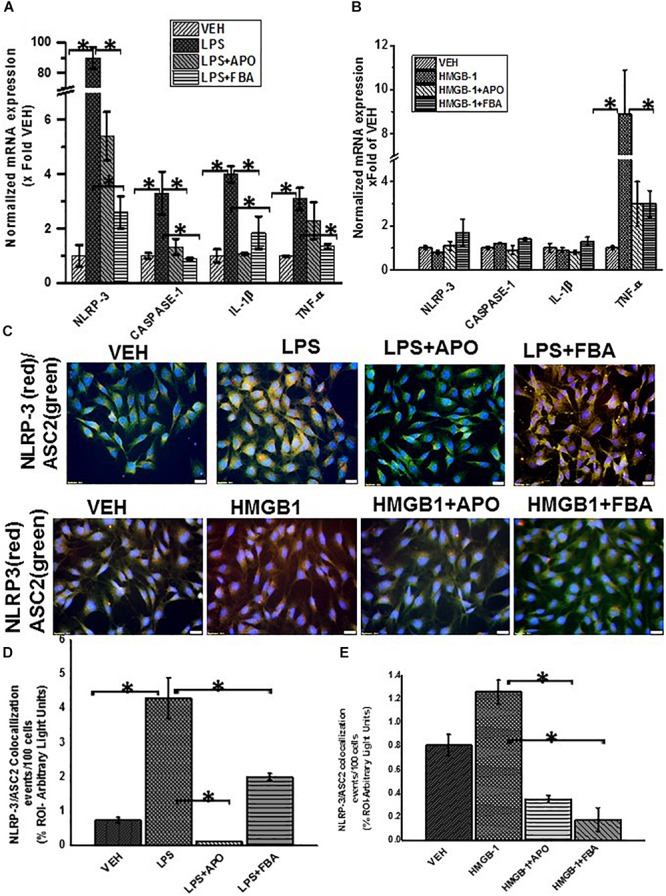
ROS mediated activation of NLRP-3 inflammasome and inflammation in EGC. **(A,B)** Quantitative real time PCR (qRT-PCR) analysis of inflammasome and inflammation (*n* = 6). mRNA expression of NLRP-3, caspase-1, IL-1β and TNF-α in rat EGC which were treated with vehicle (VEH), LPS + apocynin (LPS + APO), LPS + phenylboronic acid (LPS + FBA), HMGB-1, HMGB-1 + APO. mRNA expression is represented as a fold change of the vehicle control. Data points are represented as mean ± SEM (*n* = 3) (^∗^*P* > 0.05). **(C)** NLRP-3/ASC2 protein expression in rat EGC assessed by immunofluorescence microscopy and viewed at total magnification 400X; scale 20 μm). Colocalization events were determined for every 100 cells per field in cells treated with LPS, LPS + APO, LPS + FBA, HMGB-1, HMGB-1 + APO, HMGB1 + FBA. **(D,E)** Quantitative morphometric analysis of fluorescence intensity of NLRP-3/ASC2. Fields for morphometric analysis were randomly selected from different fields per slide and represented as% region of interest (% ROI) (^∗^*P* < 0.05).

Reactive oxygen species (ROS) can trigger activation of inflammasomes resulting in caspase 1 mediated cleavage of Il-1β and IL-18 proinflamatory cytokines (Abais et al., 2015). Our results (Figures 13A,B) showed significant increase in mRNA expression of NLRP-3, Caspase-1, IL-1β and TNF-α in LPS treated EGC but not HMGB1 treated cells which only showed an increase in TNF-α expression compared to the vehicle control (*P* > 0.05). Treatment of EGC with LPS and apocynin (LPS + APO) and FBA (LPS + FBA) significantly decreased the observed mRNA expression (*P* < 0.05).

We then investigated the protein expression of NLRP-3 and ASCII and adaptor protein of NLRP-3 in rat EGC using immunofluorescence microscopy. We found that cells treated with LPS but not HMGB1 treated cells showed a significant increase in NLRP-3 and ASCII complex formation compared to Vehicle control treated cells (VEH) indicating activation of the NLRP-3 inflammasome, when EGC encounter PAMPS. We further found that treatment of cells with LPS and FBA (LPS + FBA) showed a significant decrease in NLRP-3 protein activation (*P* < 0.05), (Figures 13C–E) suggesting the role of NOX-2 derived peroxynitrite as a candidate for the inflammasome formation.

### Increased DNA Fragmentation in Reactive Rat EGC Following Stimulation With LPS and Its Dependence on NOX-2-Induced Oxidative Stress

Increased pathogenic stimuli were found to initiate apoptosis in EGC (Macchioni et al., 2017). We also investigated the fate of these reactive EGCs when continually exposed to PAMPs or DAMPs though a tunel assay to detect fragmented DNA.

**FIGURE 14:**
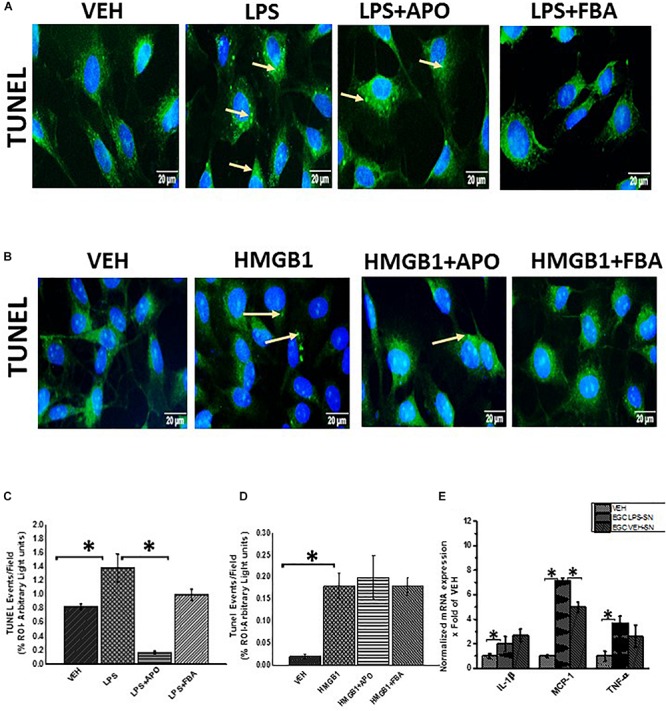
DNA fragmentation of rat EGC. **(A,B)** Tunel assay showing DNA fragmentation (*n* = 6). DNA fragmentation was determined by the Tunel assay in Vehicle (VEH), LPS, LPS + APO, LPS + FBA, HMGB-1, HMGB-1 + APO, HMGB-1 + FBA treated cells viewed at total magnification 400X; scale 10 μm. **(C,D)** Quantitative morphometric analysis of fluorescence expression of Tunel positive cells represented as Tunel events per field (^∗^*P* < 0.05). **(E)** Effect of EGC culture fluids on LPS primed intestinal epithelial cells. mRNA expression of IL-1β, MCP-1 and TNF-α in IEC-6 cells which have been primed with LPS and treated with culture fluids from EGC treated with LPS and vehicle control (*n* = 6) represented as x Fold of the vehicle control.

We found that LPS or HMGB1 treated cells showed a significant increase in co-localization events per 100 cells compared to cells treated with only vehicle control (*P* = 0.043) (Figures 14A–D). There was a significant decrease in tunel events when cells were treated with LPS and Apocynin but not FBA. And when cells were treated with HMGB1 and apocynin or FBA, there where was no significant decrease in tunel events.

### Reactive EGC Contribute to Inflammation and Intestinal Barrier Integrity in Small Intestine: Gut Decontamination by Antibiotics and Blocking EGC Immune Activation Restores Gut Barrier Protein Levels in GWI Mice

Cytokines, ROS and growth factors etc affect tight junction proteins, water channels and processes such as differentiation, apoptosis etc. (Bush et al., 1998; Bush, 2002; Yu and Li, 2014).

In this study, we showed that when LPS primed intestinal epithelial cells were treated with culture fluid from EGC, there was a significant increase in mRNA expression of proinflammatory cytokines in IEC-6 cells (Figure 14E) (*P* < 0.05). LPS primed IEC-6 cells which were treated with culture fluids from EGC treated with LPS (LPS-SN) showed a significant increase in mRNA expression of IL-1β, MCP1 and TNF-α when compared to the vehicle control (VEH) (*P* < 0.05). LPS primed cells treated with culture fluids from Vehicle control treated EGC showed a significant decrease in MCP-1 expression (*P* < 0.05) and a marked decrease in TNF-α but no significant decrease in IL-1β expression compared to the LPS primed IEC-6 cells treated with culture fluids from LPS treated EGC.

**FIGURE 15:**
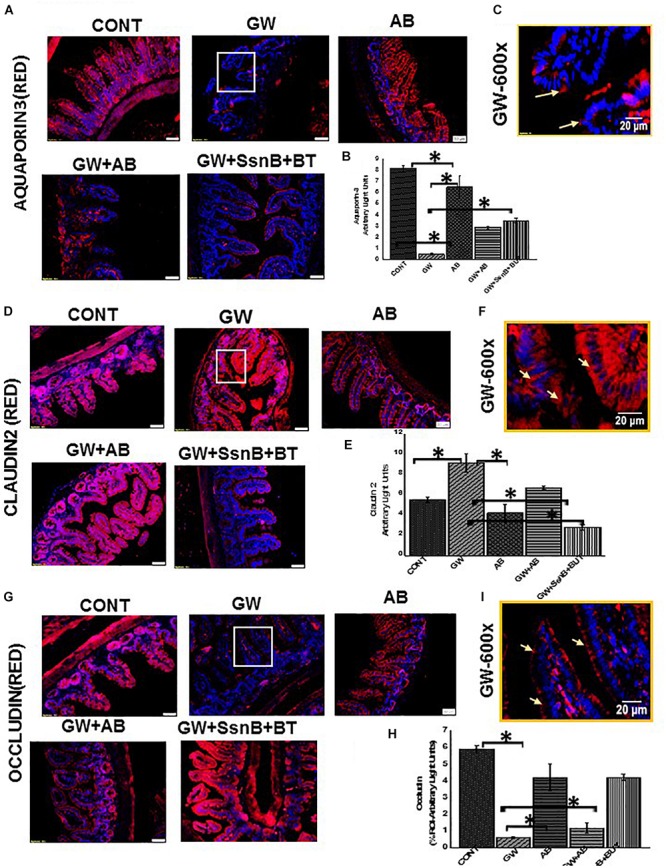
Expression of claudin 2, occludin and aquaporin 3 in mouse small intestine. **(A,D,G)** Protein expression of occludin, claudin 2 and aquaporin 3 in mouse small intestine was determined by immunofluorescence microscopy and visualized at total 200X; scale 50 μm in tissues obtained from mice treated with vehicle control (CONT, *n* = 6); mice treated with GW chemicals (GW, *n* = 9) mice co-exposed with GW chemicals and antibiotics (GW + AB, *n* = 9) and mice treated with GW chemicals, Sparstolonin B (SsnB) and Sodium butyrate (GW + SsnB + BT) (*n* = 6). **(C,F,I)** Higher magnification images for GW chemical treated group (GW) focusing on the apical or apical-lateral membranes total magnification 600X; scale 20 μm). **(B,E,H)** Quantitative morphometric analysis of immunoreactivity of occludin or aquaporin 3 is represented as (% ROI) (^∗^*P* < 0.05).

To ensure that EGC immune activation via an altered microbiome plays a significant role in gut barrier protein expression in the intestine, we studied the GW chemical treated mice for their protein levels of aquaporin, a selective water channel, occludin and claudin-2. Results showed that administration of antibiotics was associated with significantly restored the levels of aquaporin 3 in the intestine of GWI-treated mice when compared to GW-treatment (Figures 15A–C). Levels of occludin were also restored when compared to controls but were significantly elevated when compared to GW-mice only (Figures 15D–F). Claudin-2 levels have been found to be increased in association with gut integrity loss. Our results showed that use of antibiotics significantly decreased the levels of claudin-2 in antibiotic treated mice when compared to GWI-mice alone (Figures 15G–I).

To show that EGC immune activation as a result of TLR4 and specific inflammation was responsible in part in causing differential expression of tight junction proteins that may play a significant role in gut barrier protein integrity loss, we chose to use two significant compounds that has been studied for their TLR-antagonism (SSnB) and anti-inflammatory properties (Sodium Butyrate-BT) specifically in the intestine. Results showed that a combined use of TLR4 antagonist and butyrate markedly increased aquaporin levels (Figure 15A) in the small intestine when compared to GW-mice while levels of Claudin-2 were significantly decreased in the small intestine following SSnB + BT administration when compared to the same group (Figure 15D). Occludin which is decreased in GW mice and plays a significant role in maintaining gut barrier integrity was also restored to normal levels in the diseased mice following administration of SSnB + BT (Figure 15G). The results suggested that blocking TLR4 and subsequent immunoactivation that resulted in a reactive EGC phenotype in mice due to dysbiosis can be reversed by the use of these antagonists. Also, the results show that reactive EGCs might have a significant role in causing gut barrier dysfunction following activation via release of PAMPs and DAMPs and can be a cause of symptom persistence in GWI.

### Reactive EGC Modulate Tight Junction Proteins and Aquaporins in Intestinal Epithelial Cells

**FIGURE 16:**
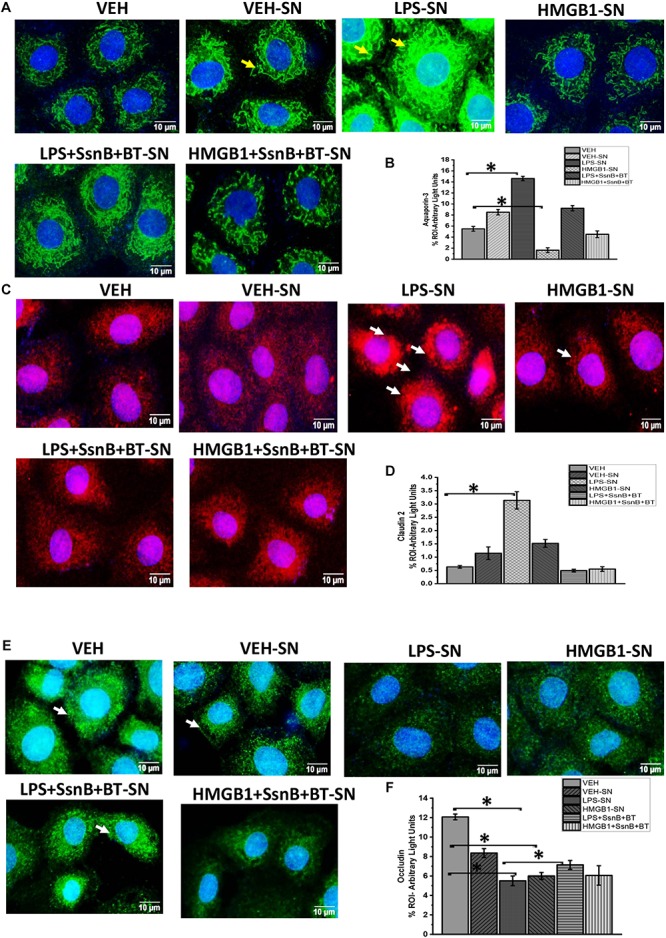
Protein expression of aquaporin 3, claudin 2 and occludin in intestinal epithelial cells. **(A,C,E)** Protein expression of aquaporin-3, claudin-2, and occludin in IEC 6 cells treated with culture fluids from Vehicle (VEH-SN), LPS (LPS-SN), HMGB1 (HMGB1-SN) and inhibitors SsnB and butyrate (LPS + SsnB + BT and HMGB1 + SsnB + BT) treated EGC. The expression of these proteins was studied by immunofluorescence microscopy and viewed at 400X total magnification; scale 10 μm. Yellow arrows indicate the localization of the proteins in the cell membrane. **(B,D,F)** Quantitative morphometric analysis of immunoreactivity of aquaporin, claudin 2, and Occludin represented as (% ROI) (*n* = 3, ^∗^*P* < 0.05).

**FIGURE 17:**
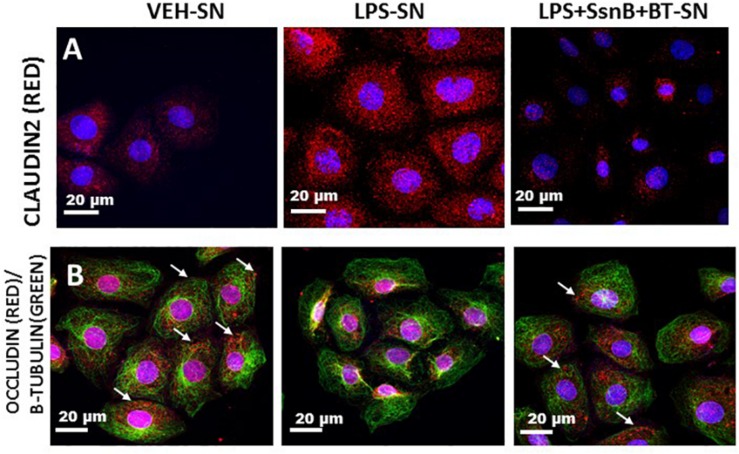
Cellular localization of Claudin 2 **(A)** and Occludin **(B)** protein expression in intestinal epithelial cells via confocal microscopy. Protein expression of Claudin 2 **(A)** or Occludin **(B)** in the cell membrane of intestinal epithelial cells treated with culture fluids from EGC treated with Vehicle (VEH-SN), LPS (LPS-SN) and LPS with SsnB and butyrate (LPS + SsnB + BT). The images were viewed under a confocal microscope at a total magnification of 630X and scale 20 μm. Occludin membrane localization was based on the tubulin staining that visualizes the outline of the cell.

We investigated the hypothesis that EGC which are exposed to DAMPS (e.g., HMGB-1) and PAMPS (e.g., LPS) modulate intestinal tight junction proteins and selective water channels by treating LPS primed IEC-6 cells with culture fluids freshly collected from EGC which have been treated with LPS (LPS-SN), HMGB1 (HMGB1-SN), vehicle (VEH-SN), LPS + SsnB + Butyrate (LPS + SsnB + BT) or HMGB1 + SsnB + Butryate (HMGB1 + SsnB + BT). Protein expression was studied by immunofluorescence microscopy observed at a total magnification of 400X; scale 10 μm. We found that expression of aquaporin-3 was significantly increased when IEC-6 cells were treated with LPS-SN, while when they were treated withHMGB1-SN the expression was significantly decreased compared to IEC 6 cells only treated with vehicle control (VEH) (*n* = 3, *p* < 0.05). IEC-6 cells treated which were treated with culture fluids from vehicle control treated EGC (VEH-SN) showed only a slight increase in aquaporin-3 protein expression, while IEC-6 cells treated with inhibitors SsnB and Butyrate together with LPS or HMGB1 restored expression of aquaporin 3 almost back to similar levels as IEC-6 cells treated with vehicle control (VEH) (Figures 16A,B). Claudin 2 expression increased significantly when IEC-6 cells were treated with culture fluids from EGC treated with LPS-SN (*n* = 3; *p* < 0.05), but only markedly, when with HMGB1 treated culture fluids (HMGB1-SN). And when IEC-6 cells were treated with culture fluids for EGC treated with SsnB and butyrate together with HMGB1 or LPS, claudin 2 levels were decreased similar to vehicle control treated cells (VEH) (Figures 16C,D). Higher magnification (630X and scale bar 20 μm) images taken under confocal microscopy are included to show the localization of this protein in the membrane (Figure 17A). IEC-6 cells that were treated with culture fluids from EGC treated with LPS (LPS-SN) or HMGB1(HMGB1-SN) showed a significant decrease in occludin protein expression compared to vehicle control treated IEC-6 cells (VEH) (Figures 16E,F). Treatment with culture fluids from EGC which had been treated with LPS or HMGB1 together with inhibitors (SsnB and butyrate) showed a restoration in occludin levels compared to the control (VEH), although the effect was stronger in IEC 6 cells treated with LPS + SsnB + BT compared to HMGB1 + SsnB + BT treated EGC culture fluids. Higher magnification (630X and scale;20 μm) images taken under confocal microscopy were used to show the localization of this protein in the membrane (Figures 17A,B). The tubulin staining used in occludin images to show the extent of occludin traversing the tubulin outline thus signifying their apical membrane localization as widely perceived (Shown by white arrows).

These results indicate that reactive EGC are strong players in modulating tight junction protein expression through production of factors that may influence gut barrier integrity.

## Discussion

Our results propose a possible molecular mechanism to explain the altered microbiome associated inflammation in a cellular level and poor gastrointestinal health which we observed in our studies on GWI (Alhasson et al., 2017; Seth et al., 2018). The results reported in this study are an advancement to our previous reported work in Gulf War illness pathology. Since gut sterility by antibiotics reversed immunopathology in GWI we used the same approach to correlate the observed gut dysbiosis with EGC immunoactivity. We found a novel role of altered microbiome in causing a reactive EGC phenotype characterized by activation of toll-like receptors, RAGE/S100B and increased expression of nitric oxide synthase. This pathway contributes to NADPH oxidase mediated generation of ROS which trigger inflammation. The mechanism of NOX2 mediated inflammation is well accepted as we and others have shown the role of peroxynitrite mediated inflammation in liver, kidney and gastrointestinal disturbances (Das et al., 2015; Alhasson et al., 2016) Further, we propose that this increased inflammation and ROS may result in enteric gliopathy and a later episodes of enteric neuropathy although this needs to be investigated further and is a speculation at this point. With continued production of proinflammatory cytokines and other destructive factors (e.g., ROS) by reactive EGC, the entire or part of the epithelial barrier in the gut might lose its integrity. This hypothesis is further strengthened by our results from the supposed blockage of TLR4 and inflammation by using SSnB and Butyrate (thus blocking EGC immune-activation), further exacerbating the observed gastrointestinal pathology in gulf war illness. This explanation not only helps us understand the acute phase of gulf war associated gastrointestinal inflammatory disorders, but also could explain why these symptoms may persist for long since a vicious cycle might exist following a continuous assault on the intestinal epithelial cells.

Enteric glial cells are important regulators of the gastrointestinal tract health. They can influence the gut microenvironment both positively or negatively depending on surrounding conditions (Capoccia et al., 2015; Ochoa-Cortes et al., 2016; Grubisic et al., 2018). Remarkably they can respond to the presence of bacteria pathogens through this TLR-RAGE/S100β-iNOS pathway. This is through the recognition of bacterial parts such as cell wall, high bacterial populations, DNA etc via toll like receptors. With an altered microbiome, there is proliferation of certain bacterial species at the expense of others and this change upsets the natural healthy balance in microbiome. This disruption happens due to several stressful stimuli e.g., infection, diet or exposure to chemicals such as in the case of gulf war illness.

Our previous studies have clearly shown that exposure to GW chemicals indeed results in altered microbiome (Alhasson et al., 2017; Seth et al., 2018) (Supplementary Figure S1). We found an increase in the Firmicutes/Bacteriodetes ratio with significant increases in several Firmicutes genera in gulf war chemical treated mice compared to the vehicle controls. Further we found an associated loss in bacteria populations such as Bacteroides, Oscillibacter and Ruminiclostridia. Increased abundance of Bacteroides for example are associated with healthy gastrointestinal states (Johnson et al., 2017), while Oscillibacter and Ruminiclostridia have also been shown to be abundant in healthy controls in studies of Crohn’s disease (Svolos et al., 2019). The decline in beneficial microbiota may have allowed for the proliferation of several bacteria populations at genus level, which usually exist in low percentages. There was a rise in several Coriobacteria, Bacilli and Verrucomicrobia bacteria. These have all been associated to increase in IBS and IBD (Distrutti et al., 2016). This upset balance of bacteria population dynamic results in normally benign bacterial populations to become pathogenic and could cause EGC to change to a reactive phenotype through toll-like receptor signaling (Zhang et al., 2015).

Both the altered microbiome and reactive EGC phenotypes have been linked to several diseased states of the gut such as IBS, IBD, gut hypersensitivity etc. (Conlon and Bird, 2014; Wang et al., 2019). However, there is scanty information concerning the true mechanism of how they contribute to these diseases. Our current study showed a correlation between altered microbiome and a reactive EGC phenotype in small intestine. Mice treated with GW chemicals (GW) had a higher expression of GFAP a protein whose increased expression has been associated with IBS, S100β/RAGE complex formation and finally an increase in nitric oxide synthase activity in EGC. These proteins were not increased in vehicle control treated mice (CONT) and their expression was significantly less in mice treated with GW chemicals and antibiotics (GW + AB). This emphasizes the role of microbiome in contributing to EGC reactive phenotype. Though we have used antibiotics to ensure gut contamination or sterility, the use of such approach may not be ensuring complete gut sterility in mice. Often the use of such antibiotics can selectively lead to bacteriostatic effects in healthy fauna while elevating the abundance of harmful bacteria in the gut. The use of germ free mice is the best approach for conducting studies where the endpoint is to assess the role gut bacteria in the pathology. Though it has to be admitted that antibiotic use for ensuring gut sterility is a standard approach where use of germ-free mice is a constraint. The use of antibiotics in our study is thus a limitation and needs further corroborative studies in future using the germ free model.

Mechanistically, we showed that the increased activation of NOX-2 following an altered microbiome and associated activation of the EGCs in GW chemical exposed mice plays a significant role in contributing to the observed intestinal inflammatory phenotype. NOX-2 in EGC and in adjacent intestinal cells, participates in oxidative stress which results from the increase in nitric oxide production. We found that the generated ROS triggered activation of the NLRP-3 inflammasome which further caused increase in inflammation and programmed cell death as showed by increased DNA fragmentation (tunel assay) in rat EGCs stimulated with LPS and/or HMGB1.

This increased inflammation and direct loss in enteric glia has been reported as in Chron’s disease (Cornet et al., 2001; Ochoa-Cortes et al., 2016). The reactive inflammatory glial phenotype is detrimental to the health of the gastrointestinal tract because it produces destructive factors which interact with surrounding cells in the intestine e.g., intestinal epithelial cells, enteric neurons etc. In our study we showed that when EGC conditioned media was applied to primed epithelial cells, there was an increase in proinflammatory cytokine expression such as IL1β, MCP-1 and TNF-α which can be conducive to a leaky gut microenvironment. Furthermore, we observed that a reactive EGC phenotype can also have detrimental effects on the EGCs as shown by increased DNA fragmentation and cell death through the increased inflammation and ROS generated. This ultimately may result in programmed cell death in glia by pyroptosis or apoptosis as shown elsewhere (Macchioni et al., 2017). The general loss in enteric glia could lead to suboptimal functioning of enteric neurons and even enteric neuropathy (Bassotti et al., 2018). This mechanism could be a possible explanation for the symptoms of GWI which continue to persist for 25 years though the present report does not study the role of an activated EGC on intestinal neurons. However, the effect of the reactive EGCs on intestinal epithelial cell barrier integrity can be profound as a blockade of the EGC activation mechanisms by SSnB and butyrate prevented protein alterations in the tight junctions. The results are also interesting since we observe a cyclical pattern of epithelial cell damage-activation of EGCs and a link to altered expression of tight junction proteins such as claudin-1,2, occludin or ZO-1 that may contribute to gut-leakiness, that eventually might fuel a continuous persistence of inflammation in the local intestinal microenvironment.

## Conclusion

We report that EGC are important players in GWI gastrointestinal disease pathology and respond to the altered microbiome in the host gut by converting to a reactive phenotype which greatly affects the healthy functioning of the gastrointestinal tract. This reactive phenotype significantly contributes to oxidative stress which further triggers inflammation, loss of gut barrier integrity and possibly death of enteric glia and enteric neurons, although further investigations need to be carried out to confirm these neuronal effects. Further, these findings provide insights into how a possible altered microbiome may be contributing to the observed GWI intestinal epithelial cell inflammatory phenotype by destabilizing the redox status of glial cells and adjacent epithelial cells via NOX-2 mediated peroxynitrite generation, inflammasome activation and release of pro inflammatory cytokines. It has to be further realized that more concrete evidence will be needed that involves germ free mice to conclude with certainty that microbiome alterations definitely dictate the observed EGC effects and therefore remains a limitation in this study. Antibiotic use for gut sterility should be overcome with more stringent experimental designs such as germ-free mouse models and gnotobiotic mice. Nevertheless, the present evidence will therefore be valuable to consider EGC nitric oxide production, formation of peroxynitrite, a redox signaling intermediate and inflammation pathways as therapeutic targets in gulf war illness.

## Data Availability Statement

The datasets generated for this study can be found in the Supplementary Material.

## Ethics Statement

The animal study was reviewed and approved by the USC Institutional Research Board (USC IACUC).

## Author Contributions

SC and DK designed and performed the experiments. DK, SS, DB, RS, YL, AM, MA, and AK performed the experiments. KS, PJ, and SL analyzed and interpreted the data. PN and MN analyzed the data and edited the manuscript. NK, KS, and RH edited the manuscript. SC and DK wrote the manuscript, edited and analyzed the data.

## Conflict of Interest

The authors declare that the research was conducted in the absence of any commercial or financial relationships that could be construed as a potential conflict of interest. The reviewer GW and handling Editor declared their shared affiliation at the time of the review.

## References

[B1] Abais J. M. Xia M. Zhang Y. Boini K. M. Li P. L. (2015). Redox regulation of NLRP3 inflammasomes: ROS as trigger or effector? *Antioxid. Redox Signal.* 22 1111–1129. 10.1089/ars.2014.5994 25330206 PMC4403231

[B2] Akimoto H. (2000). [The possible role of adhesion molecule, alpha 3 integrin, in the synthesis of intracrescentic extracellular matrix in accelerated anti-GBM nephritis]. *Nihon Jinzo Gakkai Shi* 42 1–10. 10737007

[B3] Alhasson F. Das S. Seth R. Dattaroy D. Chandrashekaran V. Ryan C. N. (2017). Altered gut microbiome in a mouse model of Gulf War Illness causes neuroinflammation and intestinal injury via leaky gut and TLR4 activation. *PLoS One* 12:e0172914. 10.1371/journal.pone.0172914 28328972 PMC5362211

[B4] Alhasson F. Dattaroy D. Das S. Chandrashekaran V. Seth R. K. Schnellmann R. G. (2016). NKT cell modulates NAFLD potentiation of metabolic oxidative stress-induced mesangial cell activation and proximal tubular toxicity. *Am. J. Physiol. Renal Physiol.* 310 F85–F101. 10.1152/ajprenal.00243.2015 26447219 PMC4675804

[B5] Bassotti G. Macchioni L. Corazzi L. Marconi P. Fettucciari K. (2018). *Clostridium* difficile-related postinfectious IBS: a case of enteroglial microbiological stalking and/or the solution of a conundrum? *Cell. Mol. Life Sci.* 75 1145–1149. 10.1007/s00018-017-2736-1 29285574 PMC11105427

[B6] Bassotti G. Villanacci V. Fisogni S. Rossi E. Baronio P. Clerici C. (2007). Enteric glial cells and their role in gastrointestinal motor abnormalities: introducing the neuro-gliopathies. *World J. Gastroenterol.* 13 4035–4041. 17696219 10.3748/wjg.v13.i30.4035 PMC4205302

[B7] Brown I. A. McClain J. L. Watson R. E. Patel B. A. Gulbransen B. D. (2016). Enteric glia mediate neuron death in colitis through purinergic pathways that require connexin-43 and nitric oxide. *Cell. Mol. Gastroenterol. Hepatol.* 2 77–91. 10.1016/j.jcmgh.2015.08.007 26771001 PMC4707972

[B8] Bush T. G. (2002). Enteric glial cells. An upstream target for induction of necrotizing enterocolitis and Crohn’s disease? *Bioessays* 24 130–140. 10.1002/bies.10039 11835277

[B9] Bush T. G. Savidge T. C. Freeman T. C. Cox H. J. Campbell E. A. Mucke L. (1998). Fulminant jejuno-ileitis following ablation of enteric glia in adult transgenic mice. *Cell* 93 189–201. 10.1016/s0092-8674(00)81571-8 9568712

[B10] Cabarrocas J. Savidge T. C. Liblau R. S. (2003). Role of enteric glial cells in inflammatory bowel disease. *Glia* 41 81–93. 10.1002/glia.10169 12465048

[B11] Capoccia E. Cirillo C. Gigli S. Pesce M. D’Alessandro A. Cuomo R. (2015). Enteric glia: a new player in inflammatory bowel diseases. *Int. J. Immunopathol. Pharmacol.* 28 443–451. 10.1177/0394632015599707 26526203

[B12] Chen Y. Liu G. He F. Zhang L. Yang K. Yu H. (2018). MicroRNA 375 modulates hyperglycemia-induced enteric glial cell apoptosis and Diabetes-induced gastrointestinal dysfunction by targeting Pdk1 and repressing PI3K/Akt pathway. *Sci. Rep.* 8:12681. 10.1038/s41598-018-30714-0 30140011 PMC6107553

[B13] Cirillo C. Sarnelli G. Esposito G. Grosso M. Petruzzelli R. Izzo P. (2009). Increased mucosal nitric oxide production in ulcerative colitis is mediated in part by the enteroglial-derived S100B protein. *Neurogastroenterol. Motil.* 21:1209-e112. 10.1111/j.1365-2982.2009.01346.x 19558426

[B14] Coker W. J. Bhatt B. M. Blatchley N. F. Graham J. T. (1999). Clinical findings for the first 1000 Gulf war veterans in the Ministry of Defence’s medical assessment programme. *BMJ* 318 290–294. 10.1136/bmj.318.7179.290 9924053 PMC27710

[B15] Conlon M. A. Bird A. R. (2014). The impact of diet and lifestyle on gut microbiota and human health. *Nutrients* 7 17–44. 10.3390/nu7010017 25545101 PMC4303825

[B16] Cornet A. Savidge T. C. Cabarrocas J. Deng W. L. Colombel J. F. Lassmann H. (2001). Enterocolitis induced by autoimmune targeting of enteric glial cells: a possible mechanism in Crohn’s disease? *Proc. Natl. Acad. Sci. U.S.A.* 98 13306–13311. 10.1073/pnas.231474098 11687633 PMC60866

[B17] Das S. Alhasson F. Dattaroy D. Pourhoseini S. Seth R. K. Nagarkatti M. (2015). NADPH oxidase-derived peroxynitrite drives inflammation in mice and human nonalcoholic steatohepatitis via TLR4-lipid raft recruitment. *Am. J. Pathol.* 185 1944–1957. 10.1016/j.ajpath.2015.03.024 25989356 PMC4483465

[B18] Distrutti E. Monaldi L. Ricci P. Fiorucci S. (2016). Gut microbiota role in irritable bowel syndrome: new therapeutic strategies. *World J. Gastroenterol.* 22 2219–2241. 10.3748/wjg.v22.i7.2219 26900286 PMC4734998

[B19] Dunphy R. C. Bridgewater L. Price D. D. Robinson M. E. Zeilman C. J. III Verne G. N. (2003). Visceral and cutaneous hypersensitivity in Persian Gulf war veterans with chronic gastrointestinal symptoms. *Pain* 102 79–85. 10.1016/s0304-3959(02)00342-1 12620599

[B20] Dyer M. R. Walker J. E. (1993). Sequences of members of the human gene family for the c subunit of mitochondrial ATP synthase. *Biochem. J.* 293(Pt 1), 51–64. 10.1042/bj2930051 8328972 PMC1134319

[B21] Gershon M. D. Rothman T. P. (1991). Enteric glia. *Glia* 4 195–204. 10.1002/glia.440040211 1827778

[B22] Grubisic V. Gulbransen B. D. (2017). Enteric glia: the most alimentary of all glia. *J. Physiol.* 595 557–570. 10.1113/JP271021 27106597 PMC5233670

[B23] Grubisic V. Verkhratsky A. Zorec R. Parpura V. (2018). Enteric glia regulate gut motility in health and disease. *Brain Res. Bull.* 136 109–117. 10.1016/j.brainresbull.2017.03.011 28363846 PMC5620110

[B24] Hansebout C. R. Su C. Reddy K. Zhang D. Jiang C. Rathbone M. P. (2012). Enteric glia mediate neuronal outgrowth through release of neurotrophic factors. *Neural Regen. Res.* 7 2165–2175. 10.3969/j.issn.1673-5374.2012.028.001 25538736 PMC4268714

[B25] Hernandez S. Fried D. E. Grubisic V. McClain J. L. Gulbransen B. D. (2019). Gastrointestinal neuroimmune disruption in a mouse model of Gulf War illness. *FASEB J.* 33 6168–6184. 10.1096/fj.201802572R 30789759 PMC6463928

[B26] Johnson E. L. Heaver S. L. Walters W. A. Ley R. E. (2017). Microbiome and metabolic disease: revisiting the bacterial phylum Bacteroidetes. *J. Mol. Med.* 95 1–8. 10.1007/s00109-016-1492-2 27900395 PMC5187364

[B27] Koch T. R. Emory T. S. (2005). Evaluation of chronic gastrointestinal symptoms following Persian Gulf War exposure. *Mil. Med.* 170 696–700. 10.7205/milmed.170.8.696 16173212

[B28] Linan-Rico A. Turco F. Ochoa-Cortes F. Harzman A. Needleman B. J. Arsenescu R. (2016). Molecular signaling and dysfunction of the human reactive enteric glial cell phenotype: implications for GI infection, IBD, POI, neurological, motility, and GI disorders. *Inflamm. Bowel Dis.* 22 1812–1834. 10.1097/MIB.0000000000000854 27416040 PMC4993196

[B29] Macchioni L. Davidescu M. Fettucciari K. Petricciuolo M. Gatticchi L. Gioe D. (2017). Enteric glial cells counteract *Clostridium* difficile Toxin B through a NADPH oxidase/ROS/JNK/caspase-3 axis, without involving mitochondrial pathways. *Sci. Rep.* 7:45569. 10.1038/srep45569 28349972 PMC5368562

[B30] Menees S. Chey W. (2018). The gut microbiome and irritable bowel syndrome. *F1000Res* 7:F1000 FacultyRev–1029. 10.12688/f1000research.14592.1 30026921 PMC6039952

[B31] Morales-Soto W. Gulbransen B. D. (2019). Enteric glia: a new player in abdominal pain. *Cell. Mol. Gastroenterol. Hepatol.* 7 433–445. 10.1016/j.jcmgh.2018.11.005 30739868 PMC6369218

[B32] Murphy F. M. Kang H. Dalager N. A. Lee K. Y. Allen R. E. Mather S. H. (1999). The health status of Gulf War veterans: lessons learned from the Department of Veterans Affairs Health Registry. *Mil. Med.* 164 327–331. 10.1093/milmed/164.5.327 10332170

[B33] O’Callaghan J. P. Michalovicz L. T. Miller J. V. Kelly K. A. (2017). Advancing the role of neuroimmunity and genetic susceptibility in Gulf War illness. *EBioMedicine* 26 11–12. 10.1016/j.ebiom.2017.11.021 29239837 PMC5832621

[B34] Ochoa-Cortes F. Turco F. Linan-Rico A. Soghomonyan S. Whitaker E. Wehner S. (2016). Enteric glial cells: a new frontier in neurogastroenterology and clinical target for inflammatory bowel diseases. *Inflamm. Bowel Dis.* 22 433–449. 10.1097/MIB.0000000000000667 26689598 PMC4718179

[B35] Palone F. Vitali R. Cucchiara S. Mennini M. Armuzzi A. Pugliese D. (2016). Fecal HMGB1 reveals microscopic inflammation in adult and pediatric patients with inflammatory bowel disease in clinical and endoscopic remission. *Inflamm. Bowel Dis.* 22 2886–2893. 10.1097/MIB.0000000000000938 27755215

[B36] Palone F. Vitali R. Trovato C. M. Montuori M. Negroni A. Mallardo S. (2018). Faecal high mobility group box 1 in children with celiac disease: a pilot study. *Dig. Liver Dis.* 50 916–919. 10.1016/j.dld.2018.04.003 29709462

[B37] Rosenbaum C. Schick M. A. Wollborn J. Heider A. Scholz C. J. Cecil A. (2016). Activation of Myenteric Glia during acute inflammation *in vitro* and *in vivo*. *PLoS One* 11:e0151335. 10.1371/journal.pone.0151335 26964064 PMC4786261

[B38] Seth R. K. Kimono D. Alhasson F. Sarkar S. Albadrani M. Lasley S. K. (2018). Increased butyrate priming in the gut stalls microbiome associated-gastrointestinal inflammation and hepatic metabolic reprogramming in a mouse model of Gulf War Illness. *Toxicol. Appl. Pharmacol.* 350 64–77. 10.1016/j.taap.2018.05.006 29751049 PMC6121708

[B39] Sharkey K. A. (2015). Emerging roles for enteric glia in gastrointestinal disorders. *J. Clin. Invest.* 125 918–925. 10.1172/JCI76303 25689252 PMC4362226

[B40] Steinkamp M. Gundel H. Schulte N. Spaniol U. Pflueger C. Zizer E. (2012). GDNF protects enteric glia from apoptosis: evidence for an autocrine loop. *BMC Gastroenterol.* 12:6. 10.1186/1471-230X-12-6 22251670 PMC3298702

[B41] Svolos V. Hansen R. Nichols B. Quince C. Ijaz U. Z. Papadopoulou R. T. (2019). Treatment of active Crohn’s disease with an ordinary food-based diet that replicates exclusive enteral nutrition. *Gastroenterology* 156 1354.e6–1367.e6. 10.1053/j.gastro.2018.12.002 30550821

[B42] Turco F. Sarnelli G. Cirillo C. Palumbo I. De Giorgi F. D’Alessandro A. (2014). Enteroglial-derived S100B protein integrates bacteria-induced Toll-like receptor signalling in human enteric glial cells. *Gut* 63 105–115. 10.1136/gutjnl-2012-302090 23292665

[B43] von Boyen G. B. Schulte N. Pfluger C. Spaniol U. Hartmann C. Steinkamp M. (2011). Distribution of enteric glia and GDNF during gut inflammation. *BMC Gastroenterol.* 11:3. 10.1186/1471-230X-11-3 21235736 PMC3034687

[B44] Wang P. Du C. Chen F. X. Li C. Q. Yu Y. B. Han T. (2016). BDNF contributes to IBS-like colonic hypersensitivity via activating the enteroglia-nerve unit. *Sci. Rep.* 6:20320. 10.1038/srep20320 26837784 PMC4738267

[B45] Wang Z. Xu C. M. Liu Y. X. Wang X. Q. Zhang L. Li M. (2019). Characteristic dysbiosis of gut microbiota of Chinese patients with diarrhea-predominant irritable bowel syndrome by an insight into the pan-microbiome. *Chin. Med. J.* 132 889–904. 10.1097/CM9.0000000000000192 30958430 PMC6595763

[B46] White R. F. Steele L. O’Callaghan J. P. Sullivan K. Binns J. H. Golomb B. A. (2016). Recent research on Gulf War illness and other health problems in veterans of the 1991 Gulf War: effects of toxicant exposures during deployment. *Cortex* 74 449–475. 10.1016/j.cortex.2015.08.022 26493934 PMC4724528

[B47] Yu Y. B. Li Y. Q. (2014). Enteric glial cells and their role in the intestinal epithelial barrier. *World J. Gastroenterol.* 20 11273–11280. 10.3748/wjg.v20.i32.11273 25170211 PMC4145765

[B48] Zakirova Z. Tweed M. Crynen G. Reed J. Abdullah L. Nissanka N. (2015). Gulf War agent exposure causes impairment of long-term memory formation and neuropathological changes in a mouse model of Gulf War Illness. *PLoS One* 10:e0119579. 10.1371/journal.pone.0119579 25785457 PMC4364893

[B49] Zhang Y. J. Li S. Gan R. Y. Zhou T. Xu D. P. Li H. B. (2015). Impacts of gut bacteria on human health and diseases. *Int. J. Mol. Sci.* 16 7493–7519. 10.3390/ijms16047493 25849657 PMC4425030

[B50] Zhou Q. Verne M. L. Zhang B. Verne G. N. (2018). Evidence for somatic hypersensitivity in veterans with Gulf War Illness and gastrointestinal symptoms. *Clin. J. Pain* 34 944–949. 10.1097/AJP.0000000000000611 29570102 PMC6110965

