# Supplementary material for: Dysbiosis-Associated Enteric Glial Cell Immune-Activation and Redox Imbalance Modulate Tight Junction Protein Expression in Gulf War Illness Pathology
Source: Front Physiol. 2019 Oct 14;10:1229. doi: 10.3389/fphys.2019.01229 (PMC6802578; doi:10.3389/fphys.2019.01229)
Supplement: Supplementary file 1 [file Presentation_1.PPTX]

## Slide 1
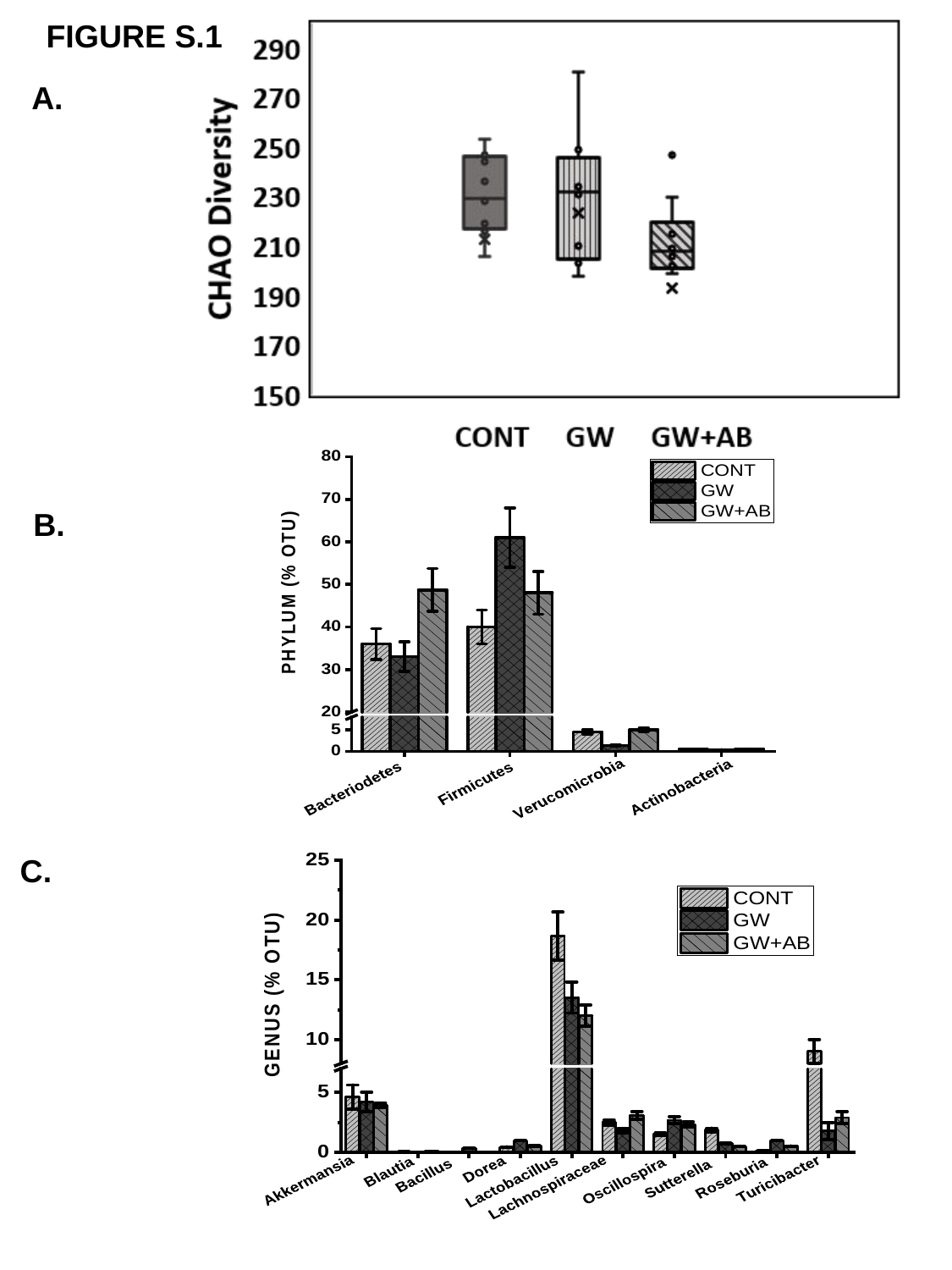

FIGURE S.1
A.
B.
C.

## Slide 2
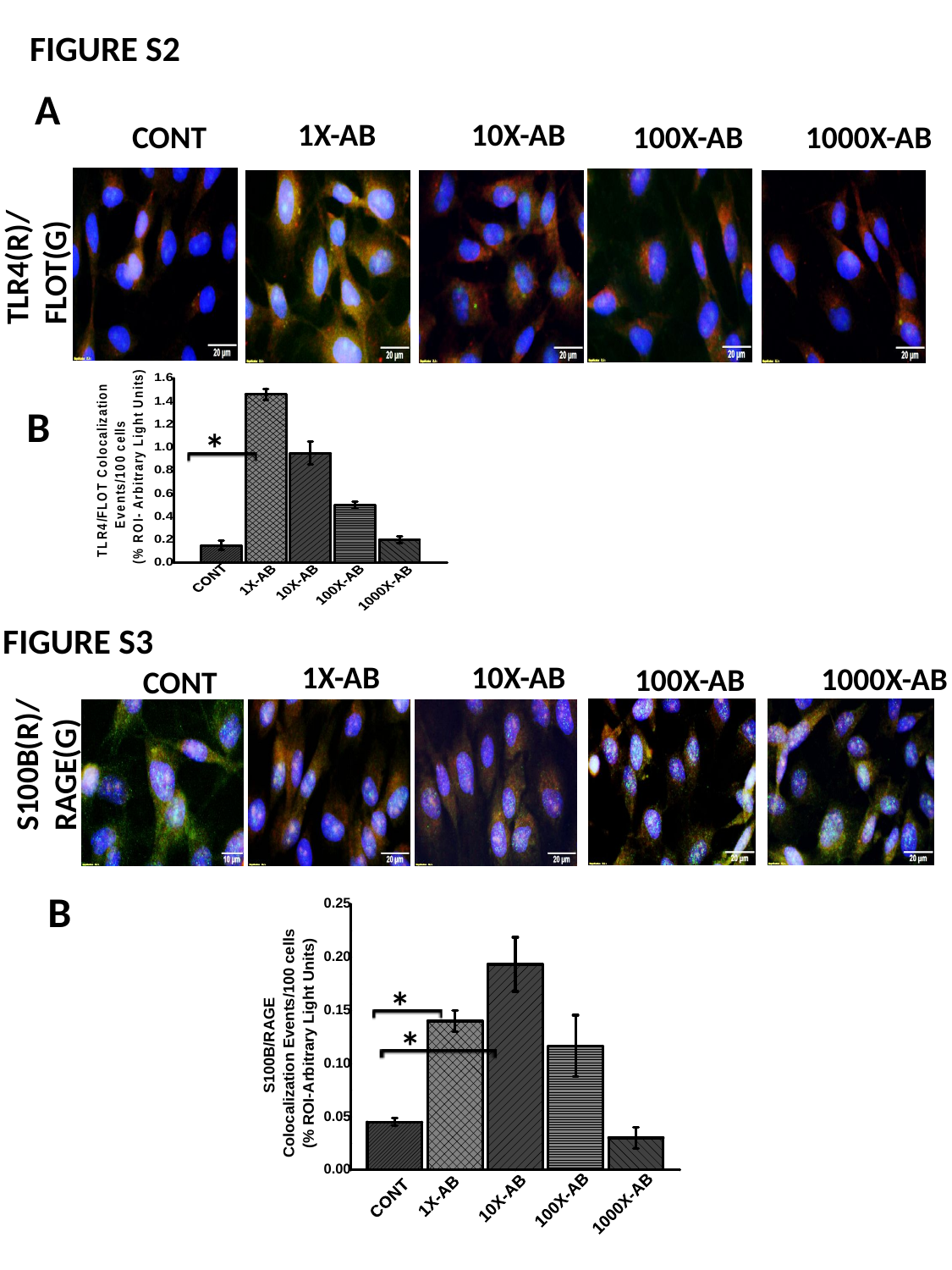

FIGURE S2
A
1X-AB
10X-AB
CONT
1000X-AB
100X-AB
TLR4(R)/
FLOT(G)
*
B
FIGURE S3
1X-AB
10X-AB
1000X-AB
100X-AB
CONT
S100B(R)/
RAGE(G)
B
*
*

## Slide 3
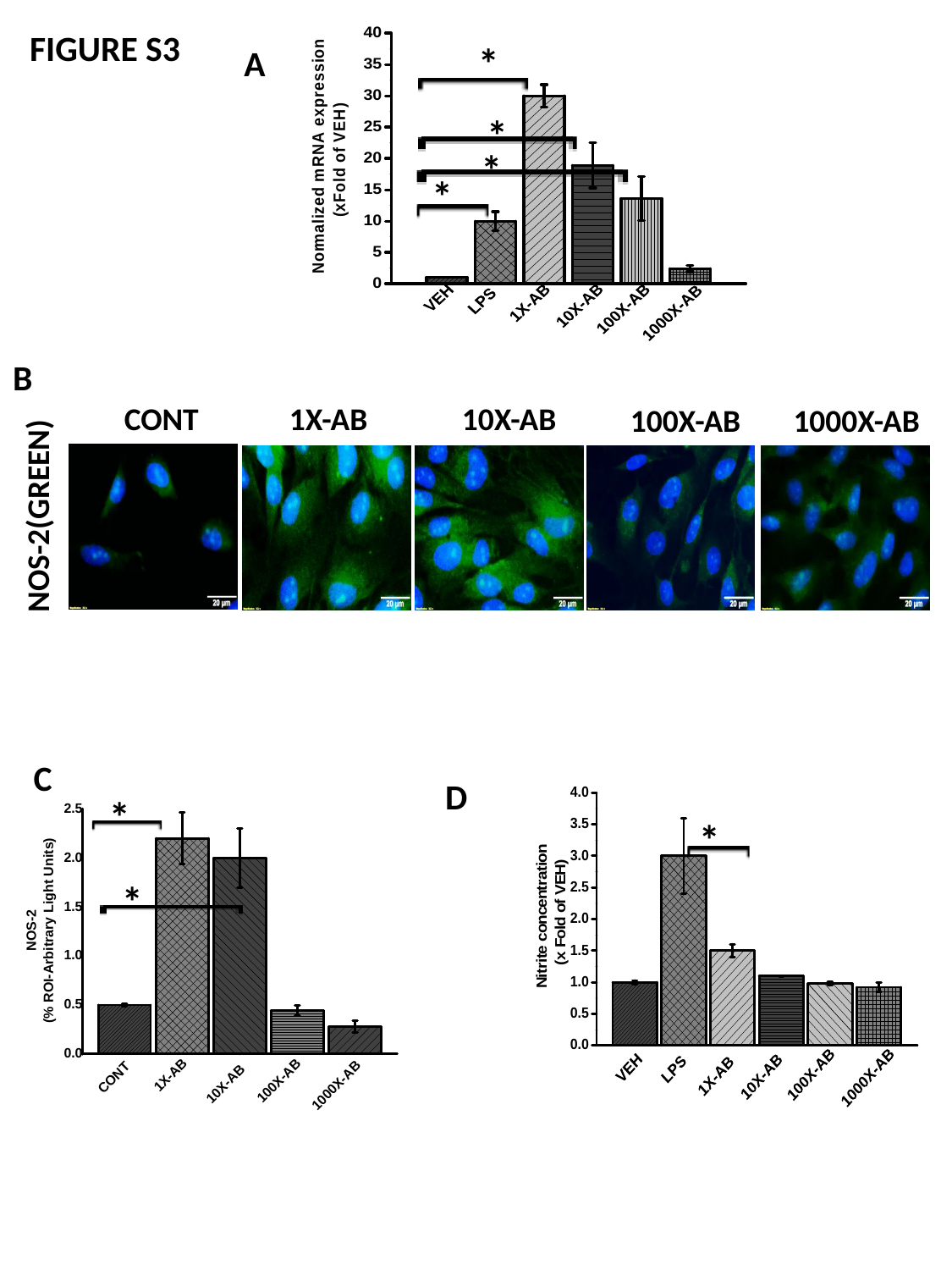

*
*
*
*
FIGURE S3
A
B
CONT
1X-AB
10X-AB
100X-AB
1000X-AB
NOS-2(GREEN)
*
C
*
*
D

## Slide 4
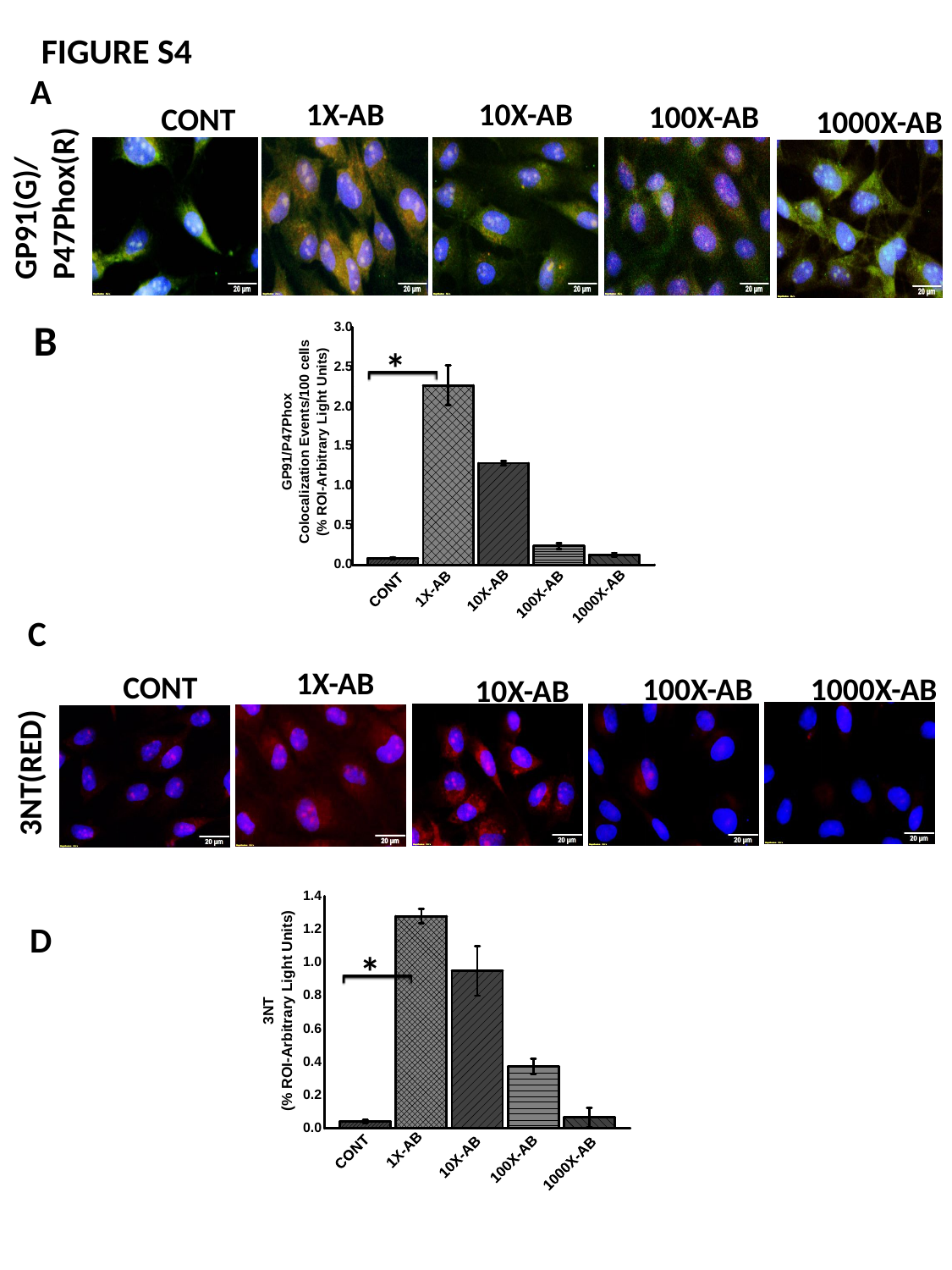

FIGURE S4
A
1X-AB
10X-AB
100X-AB
CONT
1000X-AB
GP91(G)/
P47Phox(R)
*
B
C
1X-AB
CONT
100X-AB
1000X-AB
10X-AB
3NT(RED)
*
D

## Slide 5
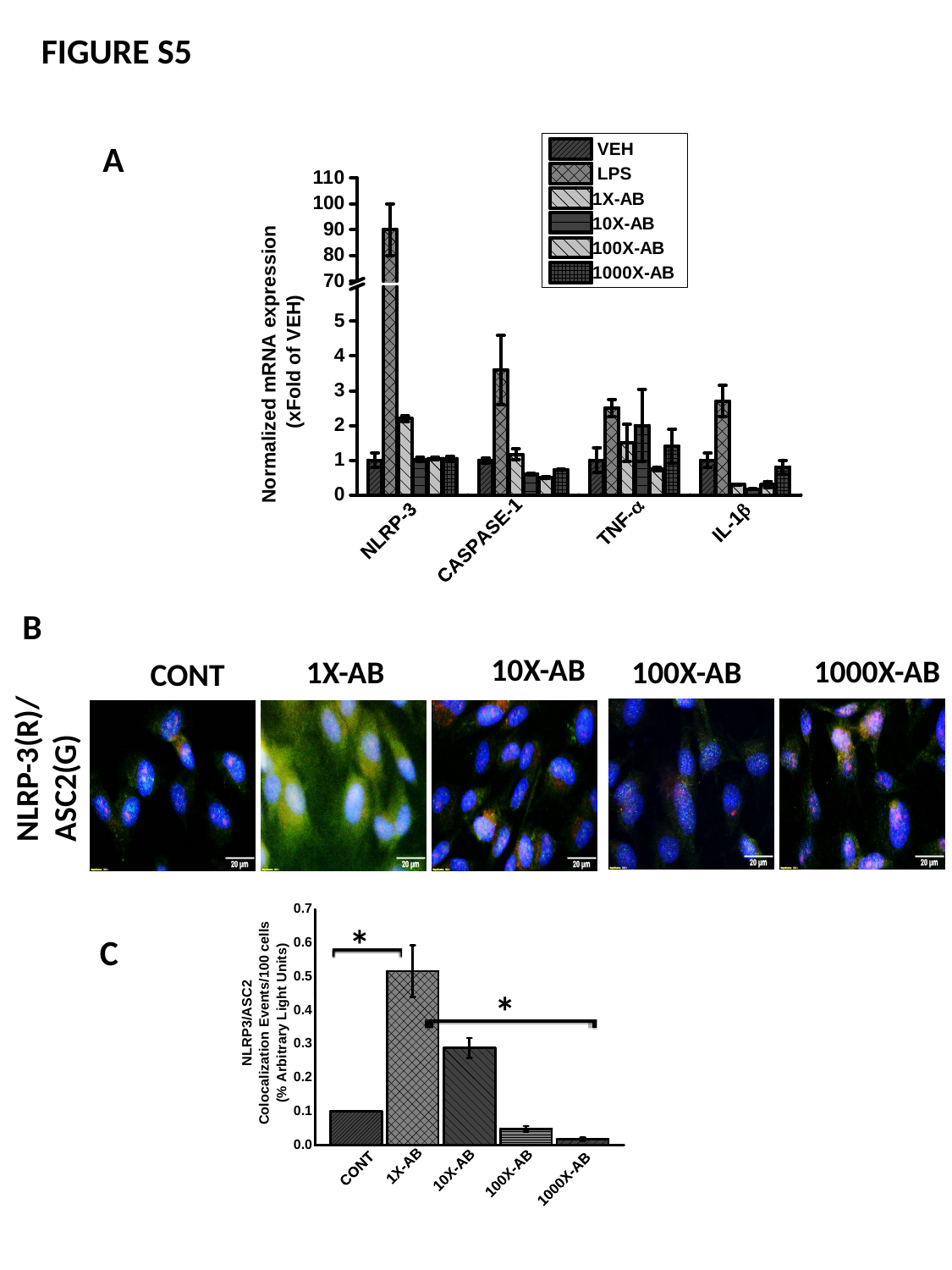

FIGURE S5
A
B
10X-AB
1000X-AB
100X-AB
1X-AB
CONT
NLRP-3(R)/
ASC2(G)
*
*
C

## Slide 6
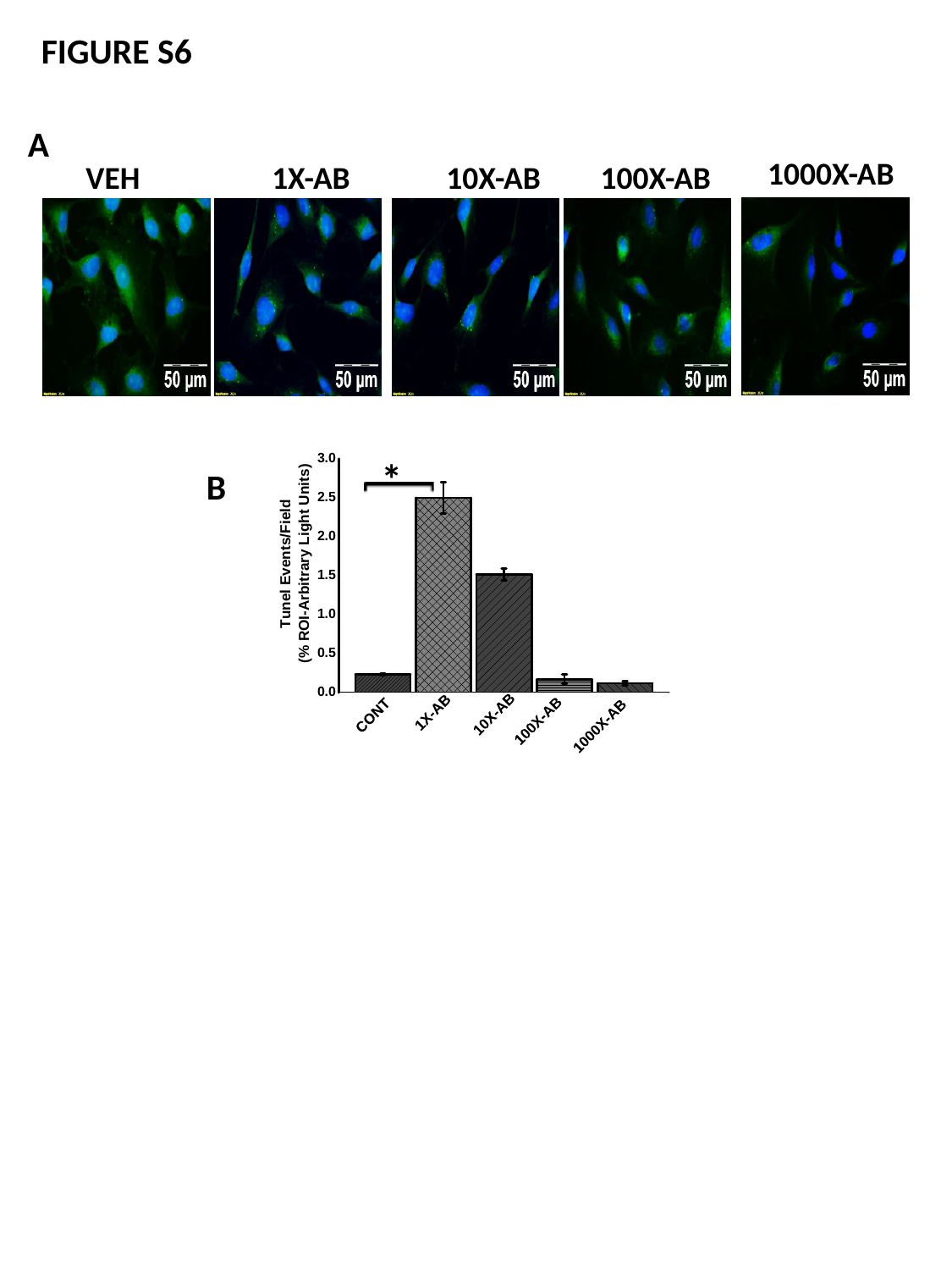

FIGURE S6
A
1000X-AB
VEH
1X-AB
10X-AB
100X-AB
*
B

## Slide 7
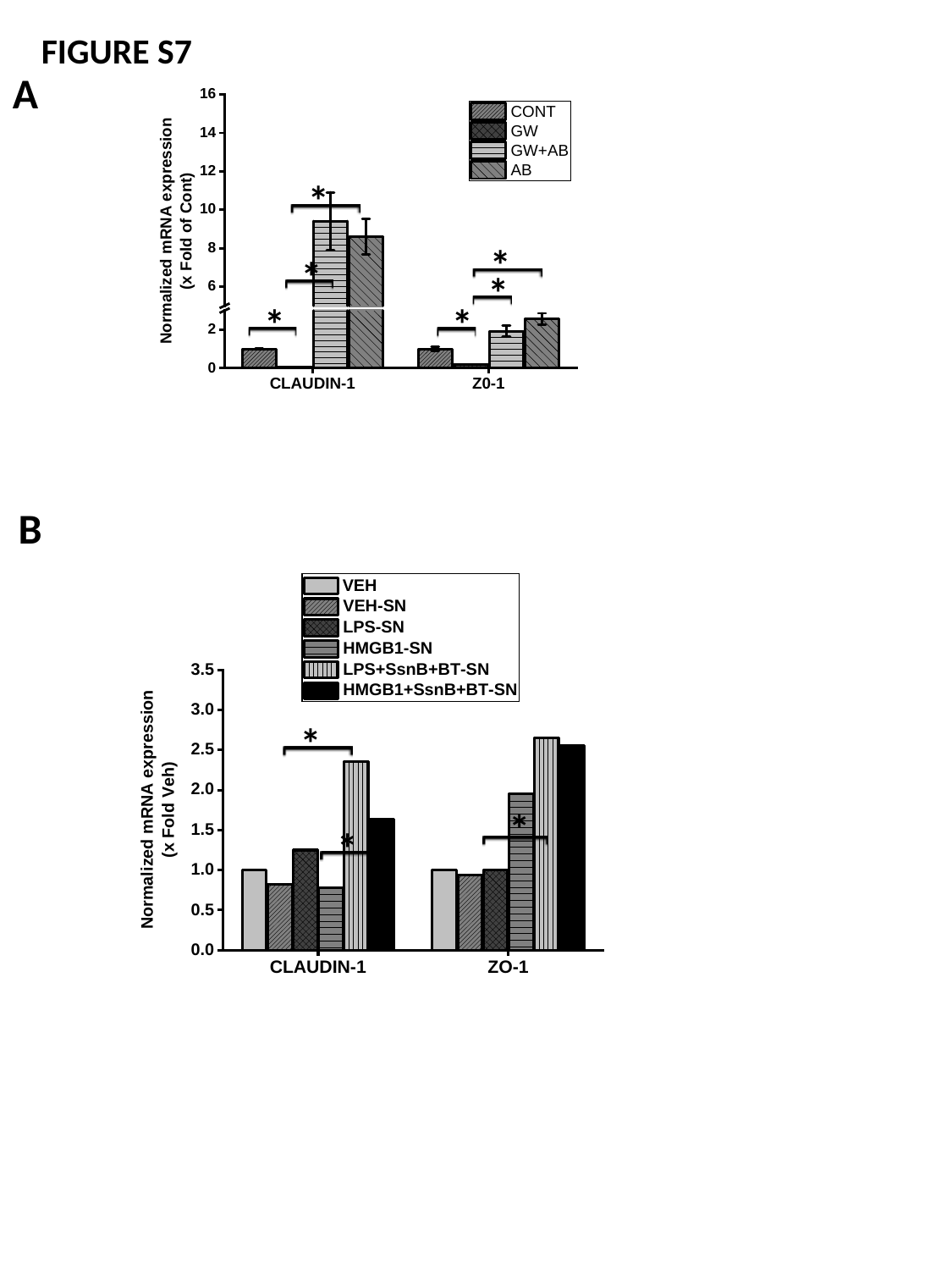

FIGURE S7
*
*
*
*
*
*
A
B
*
*
*

## Slide 8
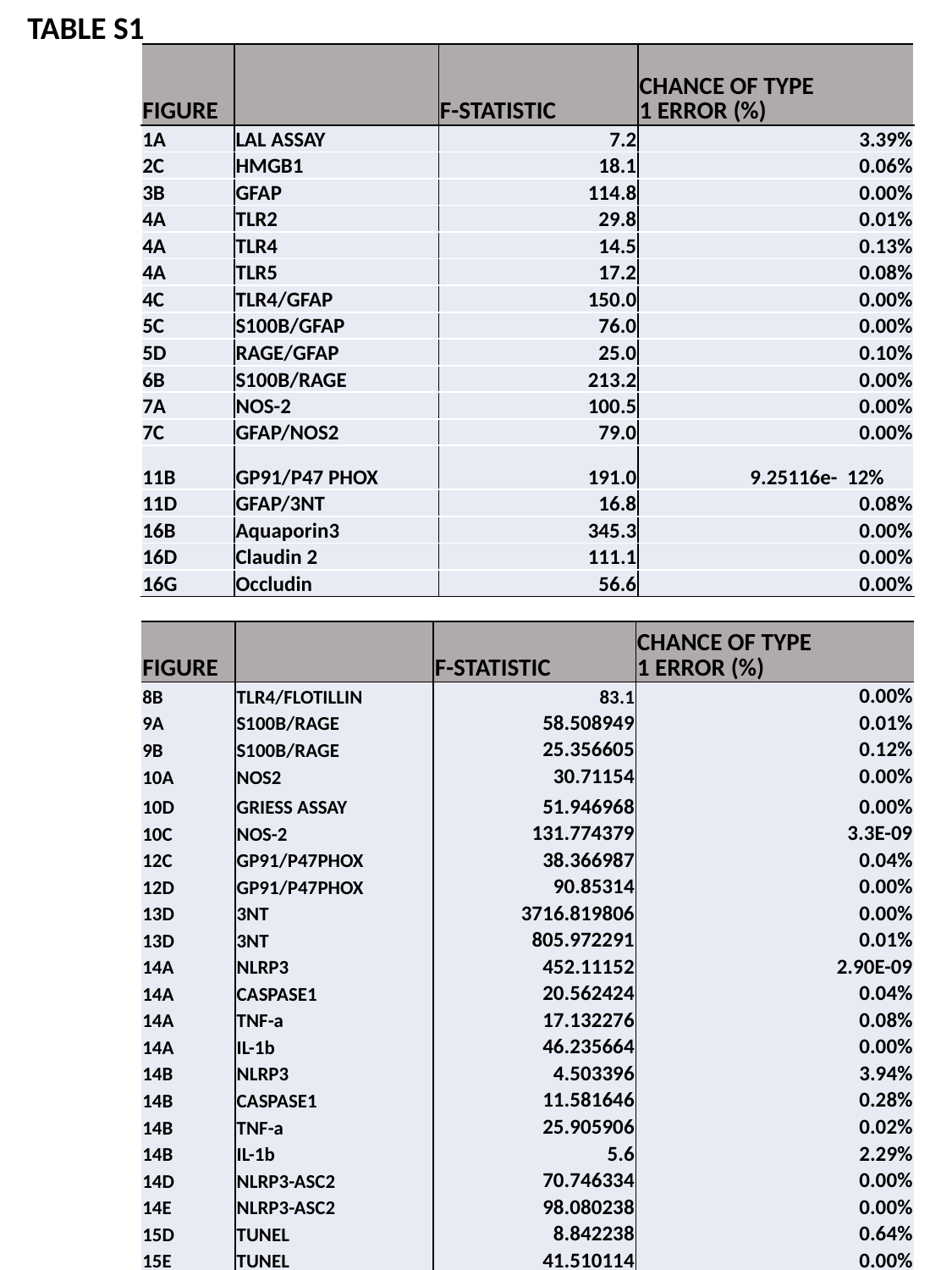

TABLE S1
| FIGURE | | F-STATISTIC | CHANCE OF TYPE 1 ERROR (%) |
| --- | --- | --- | --- |
| 1A | LAL ASSAY | 7.2 | 3.39% |
| 2C | HMGB1 | 18.1 | 0.06% |
| 3B | GFAP | 114.8 | 0.00% |
| 4A | TLR2 | 29.8 | 0.01% |
| 4A | TLR4 | 14.5 | 0.13% |
| 4A | TLR5 | 17.2 | 0.08% |
| 4C | TLR4/GFAP | 150.0 | 0.00% |
| 5C | S100B/GFAP | 76.0 | 0.00% |
| 5D | RAGE/GFAP | 25.0 | 0.10% |
| 6B | S100B/RAGE | 213.2 | 0.00% |
| 7A | NOS-2 | 100.5 | 0.00% |
| 7C | GFAP/NOS2 | 79.0 | 0.00% |
| 11B | GP91/P47 PHOX | 191.0 | 9.25116e- 12% |
| 11D | GFAP/3NT | 16.8 | 0.08% |
| 16B | Aquaporin3 | 345.3 | 0.00% |
| 16D | Claudin 2 | 111.1 | 0.00% |
| 16G | Occludin | 56.6 | 0.00% |
| FIGURE | | F-STATISTIC | CHANCE OF TYPE 1 ERROR (%) |
| --- | --- | --- | --- |
| 8B | TLR4/FLOTILLIN | 83.1 | 0.00% |
| 9A | S100B/RAGE | 58.508949 | 0.01% |
| 9B | S100B/RAGE | 25.356605 | 0.12% |
| 10A | NOS2 | 30.71154 | 0.00% |
| 10D | GRIESS ASSAY | 51.946968 | 0.00% |
| 10C | NOS-2 | 131.774379 | 3.3E-09 |
| 12C | GP91/P47PHOX | 38.366987 | 0.04% |
| 12D | GP91/P47PHOX | 90.85314 | 0.00% |
| 13D | 3NT | 3716.819806 | 0.00% |
| 13D | 3NT | 805.972291 | 0.01% |
| 14A | NLRP3 | 452.11152 | 2.90E-09 |
| 14A | CASPASE1 | 20.562424 | 0.04% |
| 14A | TNF-a | 17.132276 | 0.08% |
| 14A | IL-1b | 46.235664 | 0.00% |
| 14B | NLRP3 | 4.503396 | 3.94% |
| 14B | CASPASE1 | 11.581646 | 0.28% |
| 14B | TNF-a | 25.905906 | 0.02% |
| 14B | IL-1b | 5.6 | 2.29% |
| 14D | NLRP3-ASC2 | 70.746334 | 0.00% |
| 14E | NLRP3-ASC2 | 98.080238 | 0.00% |
| 15D | TUNEL | 8.842238 | 0.64% |
| 15E | TUNEL | 41.510114 | 0.00% |
