# Supplementary material for: Dysbiosis-Associated Enteric Glial Cell Immune-Activation and Redox Imbalance Modulate Tight Junction Protein Expression in Gulf War Illness Pathology
Source: Front Physiol. 2019 Oct 14;10:1229. doi: 10.3389/fphys.2019.01229 (PMC6802578; doi:10.3389/fphys.2019.01229)
Supplement: Supplementary file 2 [file Data_Sheet_1.docx]

**Figure S.1. Exposure to gulf war chemicals correlates with an alteration in gut microbiome population dynamics.**

**A. Diversity of bacteria populations**. Representation of most abundant bacterial populations in mice treated with vehicle control (CONT, n=9), gulf war chemicals (GW, n=9) and mice co-exposed with gulf war chemicals and antibiotics (GW+AB, n=9) to ensure gut decontamination. Treatment with antibiotics significantly decreases microbial abundance of populations compared to control and GW treated mice. Diversity of the populations is reported as CHAO Diversity. **B** and **C**. Summary of microbiome alterations in GW chemical treated mice as reported by Alhasson et al 2017 (28328972). Exposure to gulf war chemicals significantly altered bacterial populations at phylum and genus levels.

**Figure S.2. Toll-like receptor activation in rat enteric glial cells which are treated with antibiotics.** EGC were treated with a range of concentrations of the antibiotic cocktail used to decontaminate the gut (1X, 10X,100X and 1000X) to represent the possible extent of antibiotic dilution, or loss before these antibiotics reach the EGC in vivo.

There was a significant increase in TLR4 activation of TLR4 in mice treated with high concentrations of antibiotics. At 1X and 10X dilutions of antibiotics, there was significant increase in TLR4 activation, however 100X and 1000X dilutions did not result in significant TLR4 activation. Importantly, this TLR4 activation is much less compared to EGC treated with LPS or HMGB1.

**Figure S.3. S100B/RAGE complex formation in rat enteric glial cells.** There was an increase in S100B/Rage complex formation in mice treated with high concentrations of antibiotics (1X and 100X) compared to lower concentrations (100X and 1000X).

**Figure S.4. NOS-2 activation and release of nitric oxide in EGC treated with antibiotics.** EGC were treated with a range of concentrations of antibiotics (1X, 10X,100X and 1000X) to represent the possible extent of antibiotic dilution, or loss before it reaches the EGC in vivo. **A.** mRNA expression of NOS-2 in EGC: mRNA expression was determined by RTqPCR. There was a significant in crease in NOS-2 expression at all the different concentrations of EGC compared to the control**. B and C.** NOS-2 protein expression was determined by immunofluorescence, and it was found that NOS-2 was only significantly activated at very high concentrations (1X and 10X). **D.** Nitric oxide release was estimated by the Griess assay. Only a small amount of nitric oxide was released at 1X concentration of antibiotics compared to the vehicle control.

**Figure S.5. NOX-2 activation and peroxynitrite formation in EGC treated with antibiotics.** EGC were treated with a range of concentrations of antibiotics (1X, 10X,100X and 1000X) to represent the possible extent of antibiotic dilution, or loss before it reaches the EGC in vivo.

**A and B**. NOX-2 activation in EGC was determined by immunofluorescence microscopy of GP91 and P47Phox protein expression. Colocalization indicated NADPH oxidase complex formation and activation of NOX 2. There was a significant increase in the NOX-2 activation in EGC at higher concentrations of antibiotics (1X and 10X) compared to the control, but not in the 100X and 1000X concentrations of antibiotics. **C and D**. Detection of peroxynitrite formation was determined by detecting 3-nitrotyrosine in the cells which is formed when peroxynitrite free radicals react with tyrosine residues in proteins. There was an increase in 3NT formation in EGC which were treated with high concentrations of antibiotics (1X and 10X), but less EGC formed at low concentrations

**Figure S.6. Inflammasome activation in EGC treated with antibiotics.**

**A and B**. mRNA expression of NLRP3 and associated proinflammatory cytokines. mRNA expression of NLRP-3, Caspase-1, IL-1β and TNF-α was determined by RTqPCR. There was a significant increase in NLRP3 mRNA expression at 1X concentration of antibiotics, but not at other concentrations, and further, there was no significant increase in the expression of other proinflammatory cytokines at all concentrations of antibiotics. **C and D**. NLRP-3 and ASC-2 protein expression. NLRP-3 and ASC2 inflammasome complex formation was assessed by determining the protein expression levels of NLRP-3 and ASC2. Colocalizations were then determined.

**Figure S.7. DNA fragmentations in EGC treated with antibiotics.**

**A and B.** DNA fragmentation was determined by the Tunel assay which quantifies DNA fragmentation. There was a significant increase in DNA fragmentation of EGC treated with antibiotics at 1X concentration compared to the vehicle control. However, there was no significant increase in the DNA fragmentations in EGC at other concentrations of antibiotics.

**S.8**. **mRNA expression of claudin-1 and ZO-1 tight junction proteins**

**A**. mRNA expression of claudin-1 and ZO-1 in mouse small intestine, for mice treated with vehicle control (CONT), GW chemicals (GW), GW chemicals and antibiotics (GW+AB) and antibiotics only (GW) (n=3; * P<0.05). There was was a decrease in mRNA expression of claudin-1 and ZO-1 for mice which were treated with GW chemicals compared to the control, and we observed an increased expression of these proteins in mice which were treated with GW+AB or AB only mice. **B.** mRNA expression of claudin 1 and ZO-1 in IEC-6 cells which have been treated with culture fluids from EGC which had been treated with vehicle (VEH-SN), LPS (LPS-SN), HMGB1 (HMGB1-SN), HMGB1+SsnB+butyrate (HMGB1+SSNB+BT-SN) and LPS+SsnB and butyrate (LPS+SsnB+BT-SN). We saw that when EGC are treated with either LPS, they seem to produce factors that improve claudin-1 expression, but not ZO-1 expression in IEC-6 cells. And when EGC were treated with HMGB1, we see a slight decrease in ZO-1 expression but no change in claudin 1 expression in IEC 6 cells. When these EGC cells are treated with LPS+SsnB+BT or HMGB1+SsnB+BT, they seem to produce factors that increase claudin 1 and ZO-1 expression in IEC 6 cells. From these results we can see that indeed EGC produce factors that modulate different tight junction protein expression either negatively or positively, although further studies are required to confirm these results.

**Table S.1. F statistic for all experiments.**

The F-statistic was determined after a one-way ANOVA the chance of a type 1 error (p value) was also reported. The null hypothesis was rejected when the F statistic was large, and p value was very small.
